# Enhancement of PVDF membranes using graphene oxide nanoparticles and polyvinyl alcohol for water purification applications

**DOI:** 10.1038/s41598-025-23205-6

**Published:** 2025-11-13

**Authors:** Mohamed Dawam, Fatma Mohamed El-Sayed, Mahmoud Y. Zorainy, Hussein Oraby, Mohamed Gobara

**Affiliations:** 1 Department of Chemical Engineering, Military Technical College, Cairo, Egypt; 2 Hydro geochemistry Department, Desert Research Center, Cairo, Egypt

**Keywords:** Heavy metal removal, GONPs, PVDF, PVA, Membrane, Dip-coating, Surface modification, Chemical ecology, Chemical modification

## Abstract

Water contamination by heavy metal ions poses a significant environmental and public health challenge, necessitating the development of advanced and efficient treatment technologies. This study explores the advanced modification of thin-film composite (TFC) polyvinylidene fluoride (PVDF) membranes through cross-linking with glutaraldehyde (GA) and surface functionalization via a graphene oxide Nanoparticles (GONPs)/polyvinyl alcohol (PVA) coating using a dip-coating technique. The incorporation of GA as a cross-linking agent significantly enhanced the chemical and thermal stability of the thin-film coating, while the addition of GONPs improved the membrane’s hydrophilicity and metal ion rejection efficiency. The mechanical strength of the modified membranes exhibited a notable increase, with the tensile strength rising from 3.58 MPa to 6.15 MPa as the PVA/GONPs loading increased. The performance of the functionalized membranes in removing Mn^2+^ and Fe^2+^ ions as the main contaminants was systematically evaluated under varying GONPs loadings. Results demonstrated that for an initial metal ion concentration of 100 ppm, the modified PVDF membranes achieved a removal efficiency of 95.5% for Mn^2+^ and 94.6% for Fe^2+^ in the first filtration cycle. Even after five successive filtration cycles, removal rates remained above 60%, highlighting the membranes’ durability and sustained performance. This study presents a promising strategy for enhancing polymeric membranes, offering an efficient and scalable solution for heavy metal removal in wastewater treatment applications.

## Introduction

Heavy metal contamination of our environment, particularly in water, has grown to be a serious health concern for people and necessitates the creation of strategies to lessen its levels^1^. This is particularly the case of the contamination of runoff water which leads to surface water or groundwater pollution. Heavy metal ions are readily absorbed by living things, stable, non-biodegradable, and toxic even at low concentrations. Industries including textiles, mining, electroplating, battery production, paint, pesticides, printing, and photography contaminate water systems^2–4^.

Many studies concentrated on removing heavy metals like zinc, copper, and nickel that are more harmful to living organisms; however, more work is required to remove manganese II and iron II from contaminated waters. The maximum allowable levels of Mn(II) and Fe(II) in drinking water were set by the World Health Organization (WHO) at 0.05 mg/l and 0.3 mg/l, respectively^5^. If contaminated water contains excessive levels of iron and manganese, it can cause serious health and financial issues if it is used as drinking water.

Parkinson’s disease and other serious neurological conditions are brought on by manganese-contaminated water^6–8^.In the meantime, iron pollution results in laundry spoiling, a bad metallic taste and odor, and ultimately renders the water unfit for human consumption. Additionally, manganese and ferric bacteria work to cause pipe corrosion, which results in the deposition of dark sludge on the walls of drinking water pipes^9^. Therefore, reducing wastewater pollution by heavy metals and cleaning it in a sustainable and economical manner is the primary goal.

Heavy metal pollutants can be removed from wastewater using a variety of techniques. Traditional methods of extracting heavy metals from polluted water, such as flocculation, coagulation, and precipitation, usually face problems with cost and effectiveness^10^. Therefore, innovative and useful technologies are desperately needed to solve this problem. Membrane-based separation methods have become more and more popular in the water purification industry in recent years because they are incredibly easy to use, inexpensive, and selective. The benefit of membrane filtration is that it can run continuously, reducing the use of energy and ensuring a reliable and effective removal of pollutants^11,12^. Membrane has been classified into two major categories inorganic and polymeric membranes. Inorganic membranes are made up of ceramics such as titanium oxide (TiO_2_), aluminum oxide (Al_2_O_3_), zirconium oxide (ZrO_2_) and silicon oxide (SiO_2_)^13^. Ceramic membranes can be used under extreme pH and high temperature conditions, but high cost of ceramic membranes makes them less attractive.

Now a day’s polymeric membranes have got much attention for water treatment due to low cost, high flexibility and membrane forming properties. Polymeric membranes consist of organic polymers such as Polysulfones (PSF), Polyethersulfone (PES), Polyacrylonitrile (PAN), polyvinyl alcohol (PVA), Polypropylene (PP), Poly tetrafluroethylene (PTFE) and Polyvinylidene fluoride (PVDF)^14,15^. Researchers paid much attention on polyvinylidene fluoride (PVDF) polymer due to its unique properties such as inert to chemicals and oxidants, membrane forming properties, good mechanical strength and high thermal stability. Owing to these characteristic features polyvinylidene fluoride membranes have been commonly applied for water treatment^16^. Polyvinylidene fluoride membranes are hydrophobic in nature and are easily affected by fouling^17^.The hydrophobic nature of PVDF reduces the applications of polyvinylidene fluoride membranes in separation and purification of wastewater^18,19^.

Surface modification (coating of PVDF with hydrophilic polymer) is effective strategy to increase hydrophilicity of PVDF membranes. Purpose of surface modification is formation of hydrophilic layer on membrane surface which prevent the contact between membrane surface and pollutants thus diminishing fouling. Surface modification categories as physical modification and chemical modification. Chemical modification usually requires costly chemicals and special instruments which limited their practical application. Physical modification of polyvinylidene fluoride membrane can be attained by the membrane surface directly coated with hydrophilic polymer or membrane is coated by solution of chemically active monomers. Chanachai and his co-wokers researched on coating of hydrophobic PVDF membrane with chitosan by dip coating method^20^. The inorganic nanomaterials are promising modifier apart from hydrophilic polymer to minimize the fouling. The incorporation of nanomaterial into polymer matrix or on the surface has become an interesting approach for reducing fouling of PVDF membranes^21,22^.

Graphene oxide is sp^2^ oxidized derivative of graphene exhibits hydrophilic nature^23^. The presence of oxygen functional groups hydroxyl, carbonyl, epoxy and carboxyl groups at basal plane and edges impart hydrophilicity to graphene oxide. Graphene oxide used with different polymers such as polyamide, polysulfone, cellulose ester, and polyvinylidene fluoride improve thermal and mechanical properties of polymeric membranes^24^. Graphene oxide nanocomposite membranes attracted great attention for water treatment application including removal of toxic ions, water desalination and organic molecules in polluted water^25^.

 Zhao et al. incorporated 2wt% graphene oxide in polyvinylidene fluoride (PVDF) ultrafiltration rejection. Increased in pure water flux was attributed to high hydrophilicity due to the presence of abundant oxygen containing functional groups on the GO surface. These functional groups attracted water molecules inside the membrane matrix and facilitated passage of water molecules through the membranes. Rejection of BSA increases due to the formation of hydrated layer on the membrane surface and the slow change of flux ratio indicated better antifouling properties due to the introduction of hydrophilic grapheme oxide in the composite^26^.

 Zhang et al. cross-linked graphene oxide with isophoronediisocyanate (IPDI), and then coated on polyvinylidene fluoride ultrafiltration membrane by surface modification. The tendency of dye removal exceeded to 96% and heavy metal ions rejection increased to 40–70% as compared to neat PVDF membranes without the addition of graphene oxide^27^.

Akshay Modi et al. a novel nanohybrid comprising of zeolitic imidazolate framework-67 nanoparticles-decorated carboxylated graphene oxide nanosheets.

(ZIF-67/c GO) was synthesized and incorporated in polyethersulfone to enhance their separation performance. It was found that the presence of ZIF-67/c GO nanohybrid in HFMs positively altered the physicochemical properties of the resulting nanocomposite (ZcGP) HFMs, which helped them in achieving remarkably high removal of heavy metal ions (94.5 ± 1.2% for Cu^2+^ and 97.8 ± 1.1% for Pb^2+^) from the contaminated water^28^.

Synergistic effects of GO and PVP on ultrafiltration polyvinylidene fluoride membrane performance investigated by Chang et al. for the treatment of bovine serum albumin. The results showed that the membrane hydrophilicity, rejection efficiency and the antifouling performance was improved by the addition of graphene and polyvinyl pyrrolidone. It is reported that this improvement is due to the formation of hydrogen bonds between PVP and GO^29^.

Utilizing polyvinyl alcohol (PVA) to cross-link graphene oxide (GO) nanoparticles onto a membrane has been discovered to have important uses in wastewater treatment. PVA is used to add a hydrophilic group to the polymeric membrane in order to reduce membrane fouling^30^. Uniform GO dispersion in the PVA matrix enhances the mechanical properties, electrical conductivity, and thermal stability of the nanocomposite at the molecular level.

PVA is water-soluble and can easily blended or coated with GO onto PVDF membranes. PVA can act as a cross-linker or flexible polymeric bridge, improving the mechanical strength and flexibility of the modified PVDF membrane, and this is the crucial for long term durability^31^. PVA helps disperse GO more uniformly in the PVDF membrane by forming hydrogen bonds with GO’s oxygen groups, and this prevents GO aggregation and ensures a more stable and homogeneous membrane structure^32^. The hydrophilic nature of PVA reduces pore clogging and enhances water permeability. PVA helps in retaining and stabilizing GO on the membrane surface, reducing the organic fouling during membrane operation.

The goal of this work is to improve the heavy metal rejection of a TFC membrane, specifically, PVDF membranes were surface – modified by applying graphene oxide (GO). Dispersed GO in PVA was applied on the PVDF surface before using in treatment of water containing Mn(II) and Fe(II) as synthetic contaminant. GO concentration was optimized. The surface chemistry, surface Morphology, and hydrophilicity of the GO integrated membranes were also characterized at different GO loadings. The ability of the PVDF membrane to separate heavy metals was also examined using the cross flow filtration method. The influence of pH, GO loading, and zeta potential, were investigated in order to study the behavior of the TFC membranes.

## Materials and methods

### Materials

All the chemicals used in this research were analytical grade and used without any further purification. Graphite powder, sodium nitrate NaNO_3_ (purity > 98%), potassium permanganate KMnO_4_ (purity > 99%), Flat sheet of PVDF membrane (0.1 μm) and poly vinyl alcohol PVA (purity > 99%), were provided by Sigma Aldrich (USA). Glutaraldehyde (25% in H_2_O), was purchased by Sigma Aldrich(Japan).Hydrochloric acid (HCl), sodium hydroxide (NaOH), and sulfuric acid (H_2_SO_4_)were provided by Sigma Aldrich (France).Ferric chloride FeCl_3_.6H_2_O (purity > 98%), Manganese acetate (C_4_H_6_MnO_4_), Hydrogen peroxide (H_2_O_2_) were provided by Sigma–Aldrich (Australia).

### Methods

####  Preparation of graphene oxide nanoparticles

Using a modified version of Hummer’s process, graphene oxide particles (GONPs) was synthesized from graphite powder^33^. In a flask containing 23mL of concentrated H_2_SO_4_ solution, 1 g of graphite powder and 0.5 g of NaNO_3_ were added, and the mixture was then stirred in an ice bath. Subsequently, 3 g of KMnO_4_ was added gradually for 2 h. The mixture was then moved into a water bath at 35 °C and stirred for a further 30 min. Then, when the temperature increased to 98 °C, 46 mL of DI water was gradually added, and the reaction was carried out for 12 h.It was then cooled to the room temp, and 140mL of DI water were added to the mixture with stirring, then add 3mL of H_2_O_2_ (30%) to reduce the remaining KMnO_4_.Then the colure changed from brawn to bright yellow indicating the successful formation of GONPs.

**Fig. 1:**
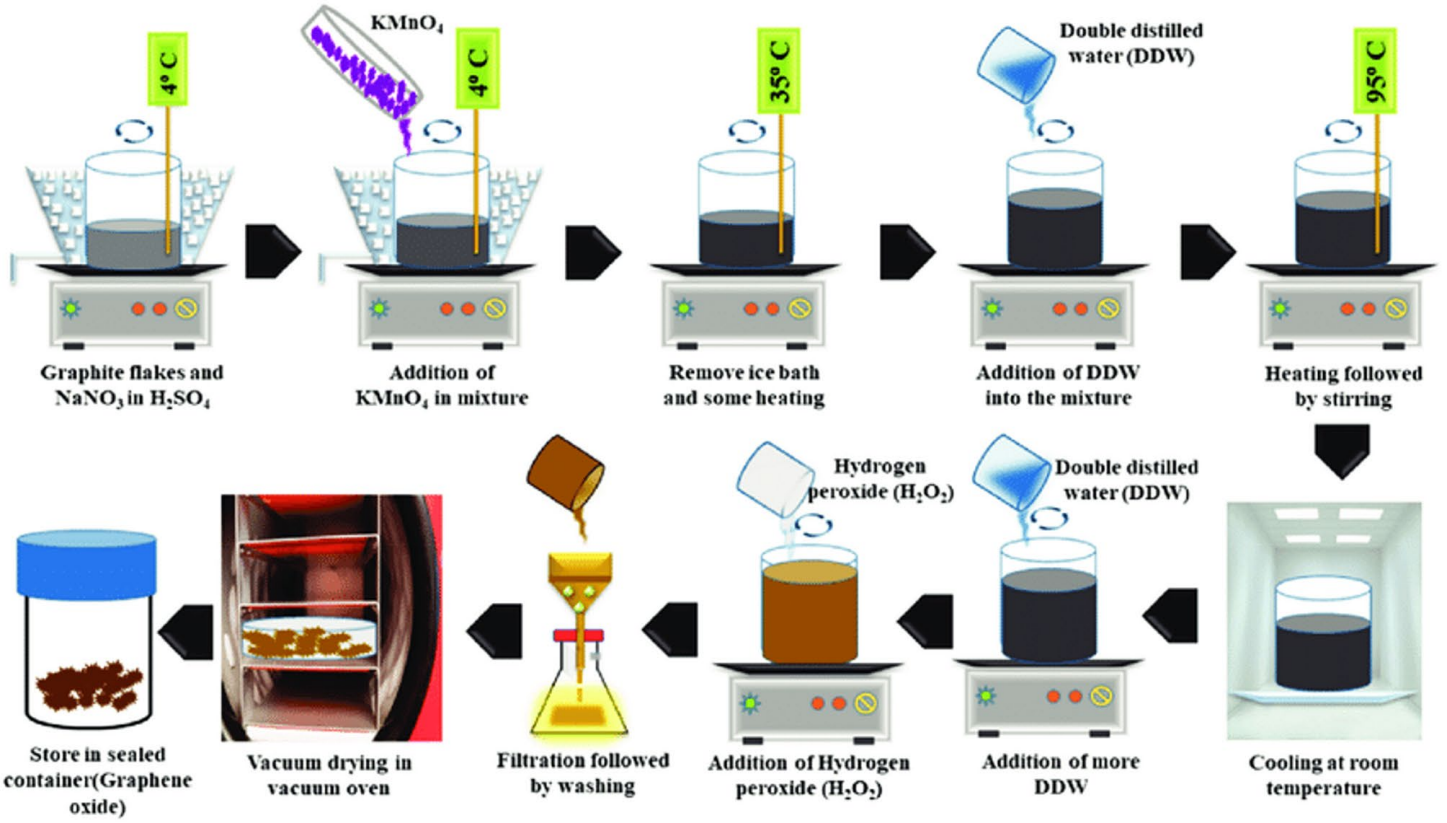
Schematic diagram for the synthesis of GONPs.

The mixture was filtered with centrifuge at 7000 rpm for 20 min. The residue was washed with 100 mL ethanol then filtered through nylon membrane filter. The resultant product of GONP was then dried in a vacuum oven at 40 °C for 24 h. The schematic diagram for the synthesis of GONPs is shown in Fig. 1.

#### surface modification of PVDF membranes using GONPs/PVA solution

**Fig. 2:**
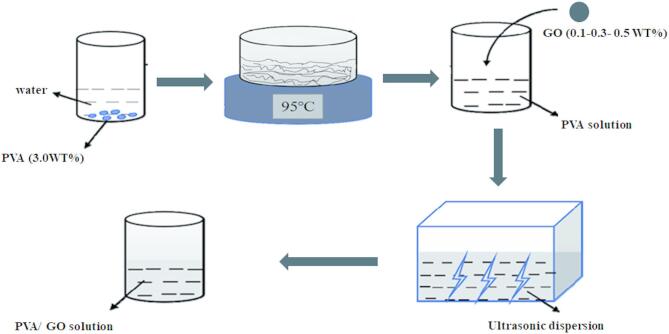
Schematic diagram for preparation of GONPs/PVA coating solution.

Selected membrane pieces which have a cross section area of 42.0 cm^2^ were washed with ethanol to remove all the dust and dirt and then dried at room temperature. First, a homogeneous PVA solution containing GONPs was prepared for thin-film coating of PVDF membrane. PVA polymer (3.0 g) was dissolved in 100 ml DI water at 100 °C for 5 h then graphene oxide nanoparticles (GONPs) (0.1, 0.3, and 0.5wt. %) were mixed with the solution. Before coating, the solution was cooled down to room temperature as seen in Fig. 2.

**Fig. 3:**
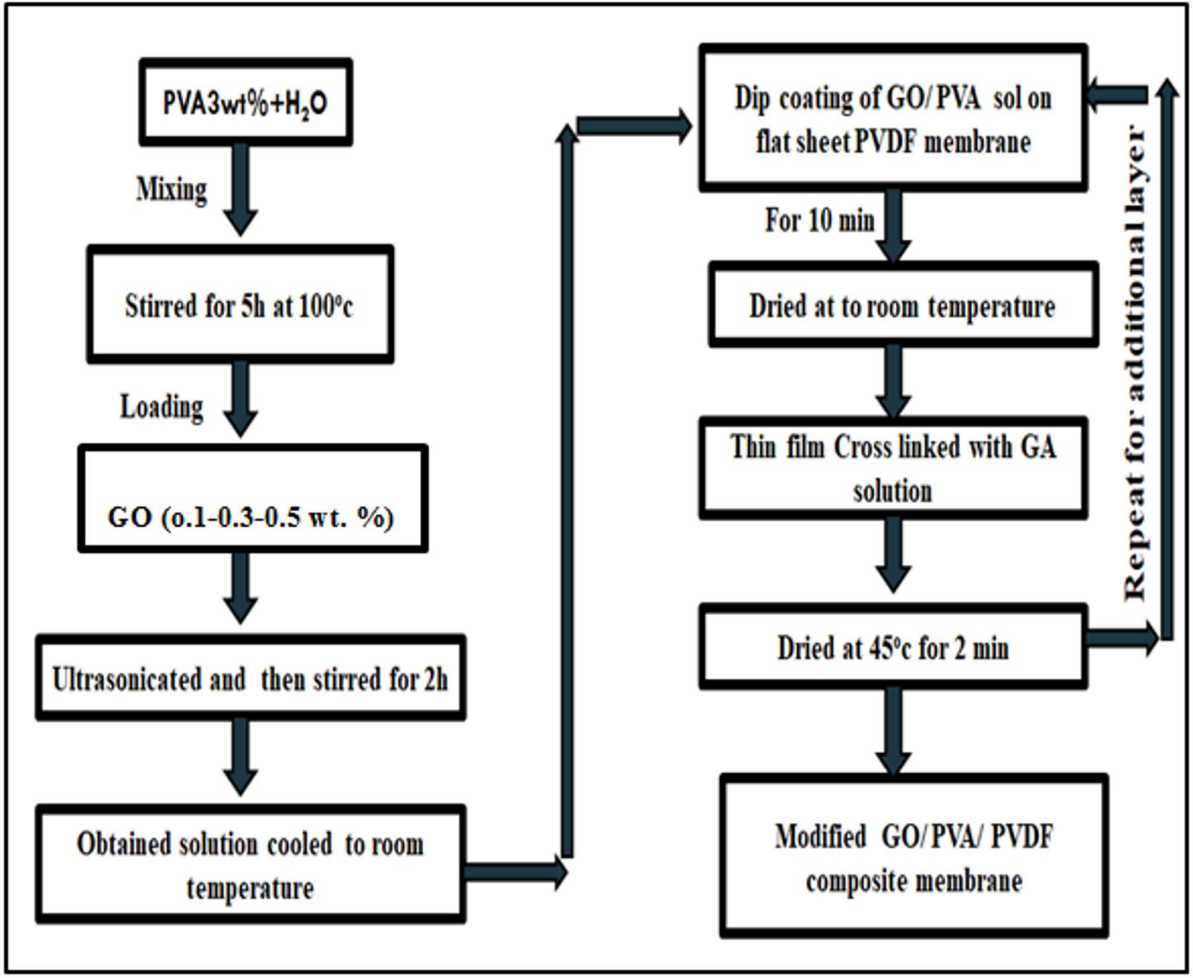
Flow diagram for the synthesis of TFC GONPs/PVA/ PVDF membrane.

The flat sheet PVDF membrane was then dipped into PVA/GONPs solution for 10 min. The excess solution was drained off by hanging the membrane pieces and then dried completely at room temperature. The flow diagram for the TFC PVDF membrane processing is shown in Fig. 3.

**Fig. 4:**
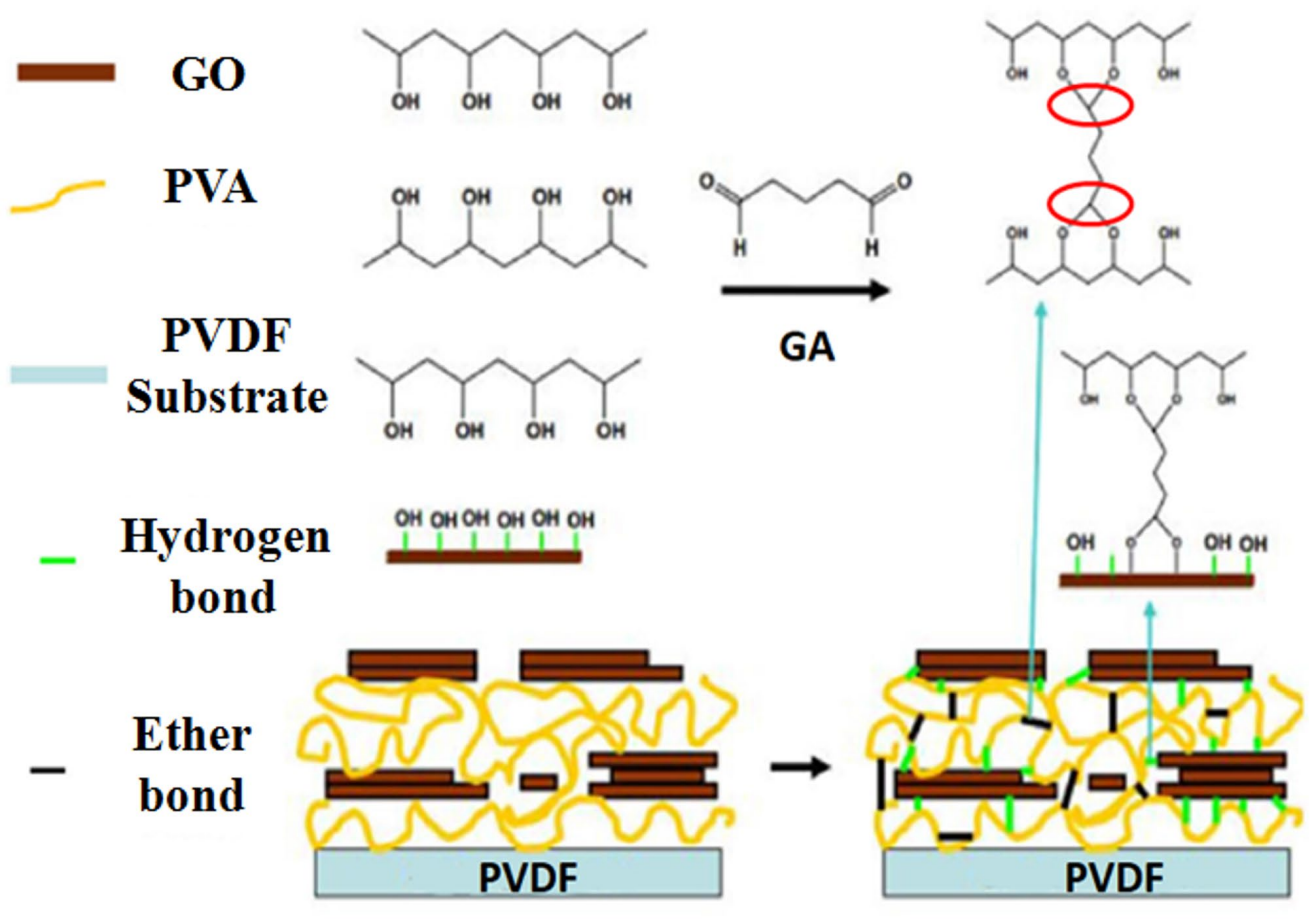
Schematic diagram of cross linking PVA/ GONPs with GA on PVDF membrane.

Completely dried coated films were then immersed in the cross-linking solution having 2 wt% of glutaraldehyde (GA) and 0.5 wt% of H_2_SO_4_ for 5 min at room temperature and 2 min at 45 °C in order to reduce the membrane swelling. Here in the GA solution, H_2_SO_4_ acted as a catalyst for the cross-linking reaction. The above procedure (coating and cross-linking) was repeated twice for each membrane to get better stability and efficacy of nanoparticles. Finally, the membranes were dried at 45 °C for 5 min and then washed with DI water to remove all the traces of cross-linking GA and dust particles^34^. Figure 4 is a schematic diagram of the PVDF membrane cross-linking PVA/GONPs with GA.

### Graphene oxide nanoparticles (GONPs) characterization

Formation of GONPs was initially investigated via high-resolution X-ray diffraction (XRD) (BRUKER-AXS, Karlsruhe, Germany) at a scanning rate of 4°/min, within the diffraction angle from 5 to 60°.The structures were further determined by Fourier transform infrared spectroscopy (FTIR) (Thermo FTIR spectrometer, Dreieich, Germany), spectra were taken in the wave number range of 4000–400 cm^− 1^ with a resolution of 4 cm^− 1^ and at least 16 scans per sample. Surface-Enhanced Raman Spectroscopy (SERS) also provides important information about the structural feature of GONPs. Surface morphology of graphite and GONPs were studied by field emission scanning electron microscope.

### Characterization of membranes

#### Surface features of the PVDF membrane

A field-emission in- lens scanning electron microscope (FEISEM) was used to analyze the surface morphology of the plain and modified PVDF membrane. The membranes were cleaned and dried, then cut into tiny small square samples (~ 1 × 1 cm^2^) and sputter-coated with gold particles to increase electrical conductivity. The images were scanned at various magnifications under high voltage in order to determine the presence and distribution of GO nanoparticles over the membrane’s surface.

#### Surface polarity measurements

An effective characterization method for studying the surface’s wettability is the contact angle measurement. Generally, a solid surface is considered hydrophilic when the water contact angle with it is less than 90° and hydrophobic when it is more than 90°. By using the sessile drop method at room temperature, the contact angle of the plain and modified PVDF membrane at varying GONPs loading in thin-film coating was determined. When the droplet landed on the dry membrane surface, Photos were taken, and the contact angles with the water were then calculated. The average of five successive measurements of the same membrane taken at various points is the reported contact angle.

#### Surface roughness measurements

Surface roughness for all the membranes was measured using an atomic force microscope (AFM) (Asylum MFP-3D Infinity). Small part of the prepared membranes (≈ 1.5 × 1.5 cm^2^) was glued on a flat and hard piece of glass. The membrane was scanned and imaged in a scan size of 2 × 2 µm^2^. Roughness parameters were obtained and reported in terms of root mean square roughness (RMS) and mean surface roughness (SA). Recorded results are the average of three measurements of a sample.

#### The membrane’s surface charge

A streaming potential analyzer (Malvern surface zeta potential) was used to measure the membrane surface Zeta potential. A tiny piece of membrane (1 × 1 mm^2^) was added to DI water at the desired pH in order to determine the membrane’s surface charge. The values given are the mean of three runs of three distinct membrane samples at pH values between 3 and 10.

#### Mechanical characteristics

A Universal Testing Machine was used to characterize the plain and modified PVDF membranes at 25 °C and 50% relative humidity. Samples of (5 × 40 mm^2^) were cut from the film, and then the two ends were fastened to the sample holders, which were spaced 30 mm apart. The two sample holders were separated during the measurement process at a consistent rate of 5 mm/min. until the sample broke. Four distinct samples were measured in order to determine film’s mechanical properties.

#### Filtration performance

**Fig. 5:**
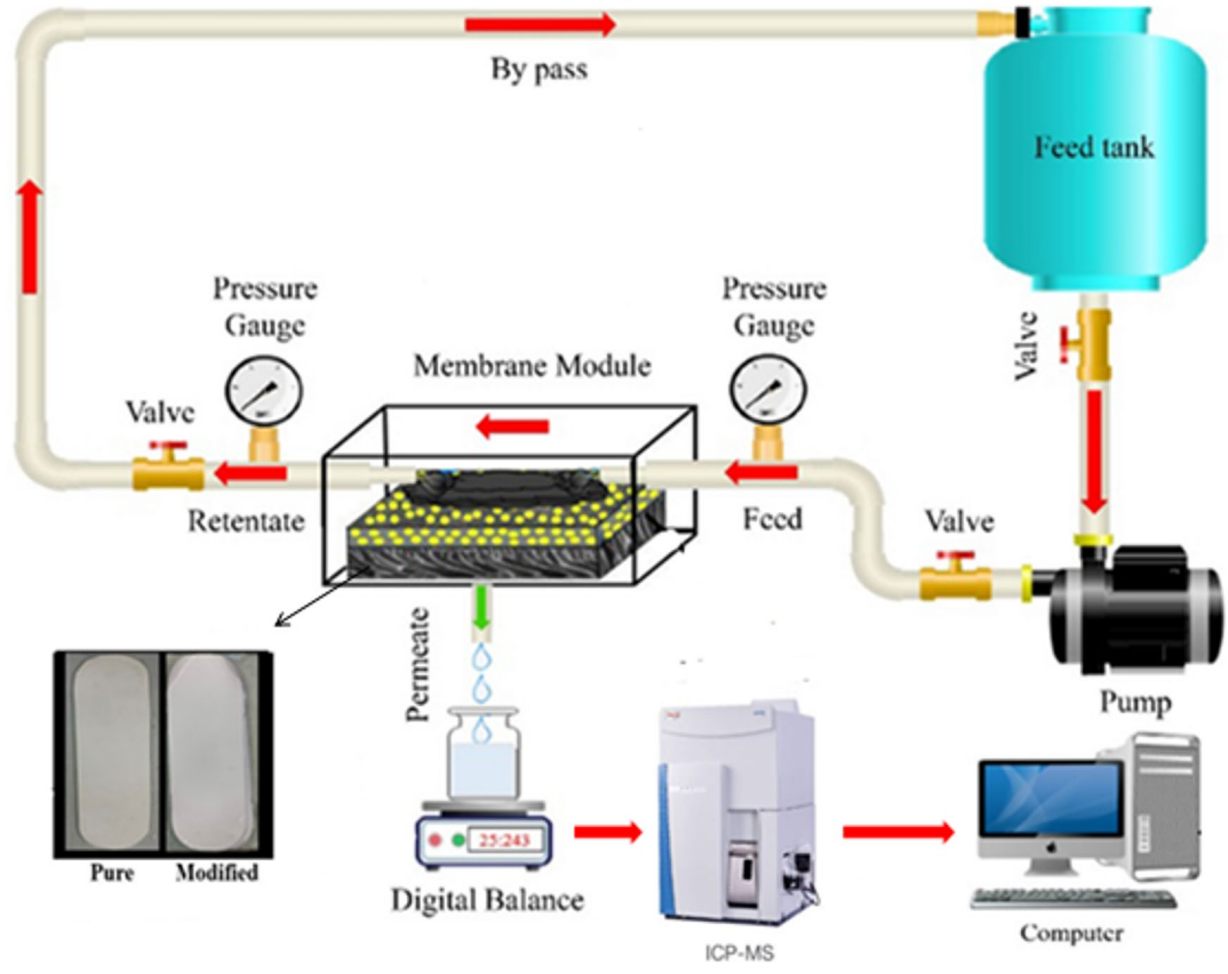
Schematic illustration of the cross-flow filter.

A cross-flow filtration system was used to evaluate the filtration capabilities of the plain and modified PVDF membranes as shown in the schematic diagram Fig. 5. All filtration experiments were conducted using an across-flow filtering system for membranes with varying GONPs loading. At an operating pressure of 3.0 bar, the flow rate was maintained at 18 L/hrs., while the membrane with an active filtration area of 42.0 cm^2^ was positioned in the cross-flow filtration unit. DI water was utilized to calculate the pure water flux (*J*) by using Eq. (1).

1 $$\:\varvec{J}=\frac{\varvec{V}}{\varvec{A}}.\varvec{t}$$

where *A* is the active membrane surface area employed for filtering and *V* is the total volume of permeate collected within time (*t*).

Prior to collecting, the refuse for analysis, the feed solution was agitated throughout the setup for a minimum of 20 min and all filtration experiments were conducted at room temperature. Using ferric chloride and manganese acetate, respectively, in 1000 ml of deionized water, stock solutions of Fe^2+^ and Mn^2+^ metal ions at 1000 ppm were made. These stock solutions were diluted to 100 ppm solutions. Using H_2_SO_4_ and NaOH solutions, the pH of the liquids was changed. Using fixed quantities of heavy metals, the batch procedure was carried out at several pH values to find the ideal pH for the best heavy metal removal by PVDF membrane. A filtering assembly was used to filter the diluted solutions through the prepared membrane while they were at room temperature. ICP mass spectroscopy was used to measure the concentration of Fe^2+^ and Mn^2+^ ions in the permeate. At regular intervals, feed and permeate samples were taken, and the change in the heavy metals concentration were measured. The removal percentage *R* (%) for each experiment was calculated using Eq. (2).

2 $$\:\varvec{R}\left(\varvec{\%}\right)=\left(\frac{\varvec{C}\varvec{i}-\varvec{C}\varvec{f}}{\varvec{C}\varvec{i}}\right).100$$

where *C*_*i*_ represents the initial concentration (measured by ICP-MS) and *C*_*f*_ represents the final concentration after a process like filtration or treatment (also measured by ICP-MS).

#### Long-term stability test of the membrane

To evaluate the stability of the PVA/GONPs-modified PVDF membranes during repeated use, long-term stability experiments were conducted. The membranes were tested over five consecutive filtration cycles, each lasting 1 h of continuous stirring and filtration at the same operating conditions (3.0 bar, 18 L/h, 200 rpm, room temperature). After each cycle, the membrane was washed thoroughly with deionized water before reuse in the next cycle. The Fe^2+^ and Mn^2+^ removal efficiencies were measured for each cycle to determine the stability and reusability of the membranes. The variation in rejection efficiency and water flux across the cycles was used as an indicator of the long-term stability of the modified PVDF membranes.

## Results and discussions

### graphene oxide nanoparticles (GONPs) characterization

#### X-Ray diffraction

**Fig. 6:**
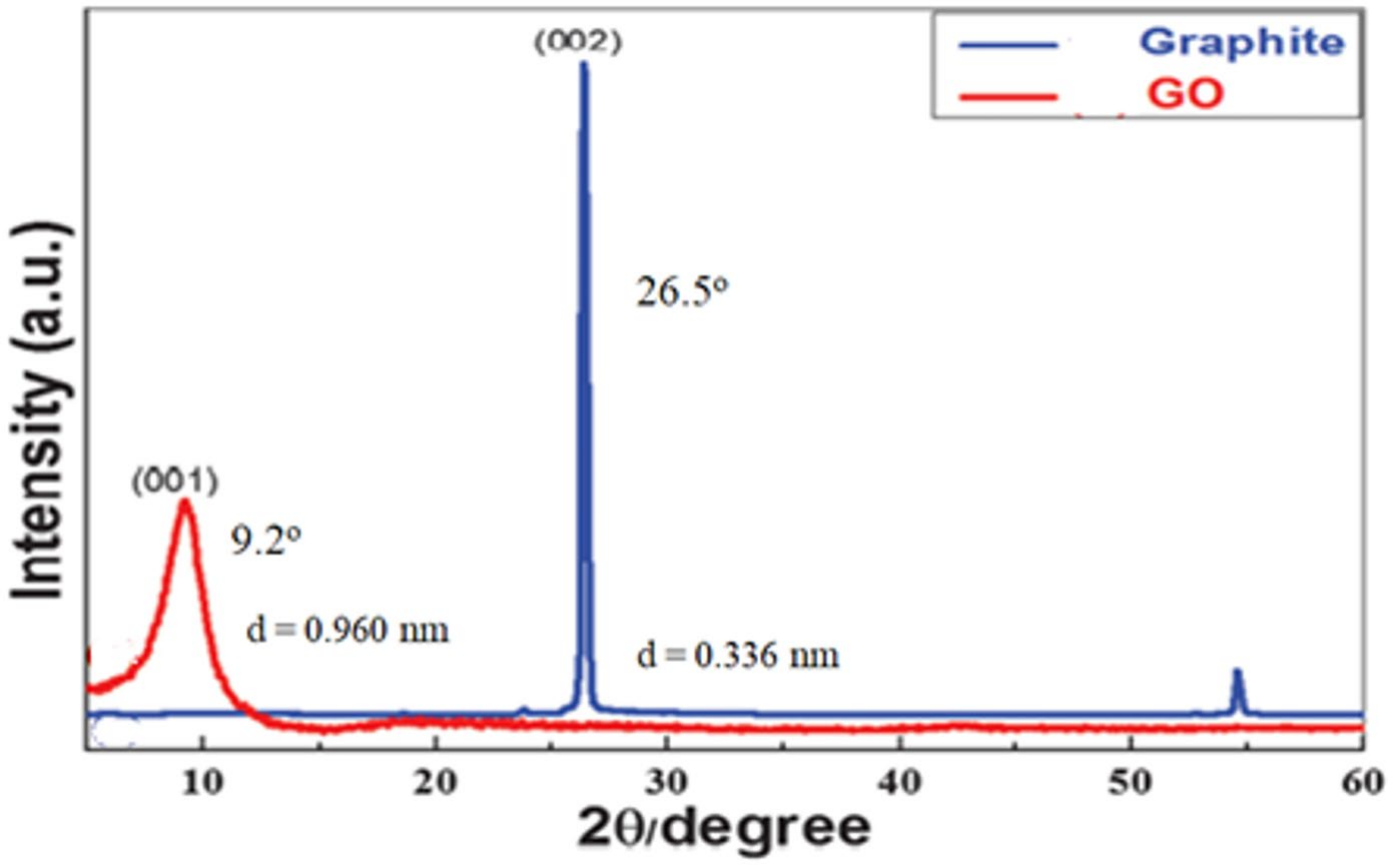
XRD spectra for graphite and graphene oxide nanoparticles.

XRD was conducted to look at the successful formation of GONPs. Figure 6 Showed the XRD patterns of graphite powder and GONPs. The graphite exhibits a peak at 2θ = 26.5° which corresponds to the reflection of the plane (002), while for the GOPs the peak appears at 2θ = 9.2°, and corresponds to the reflection of the plane (001)^35,36^.

The increase in the interplanar distance of the GONPs in comparison with the graphite from 0.336 nm to 0.96 nm due to the presence of functional oxygen groups introduced by the oxidation of the graphite. The characteristic spacing of the GONPs generally ranges between 0.7 and 0.8 nm but may vary slightly at higher or lower values, depending on the degree of functionalization^37,38^. These results confirm that the GONPs were synthesized from neat graphite via previously stated procedure.

#### Fourier-transform infrared spectroscopy

**Fig. 7:**
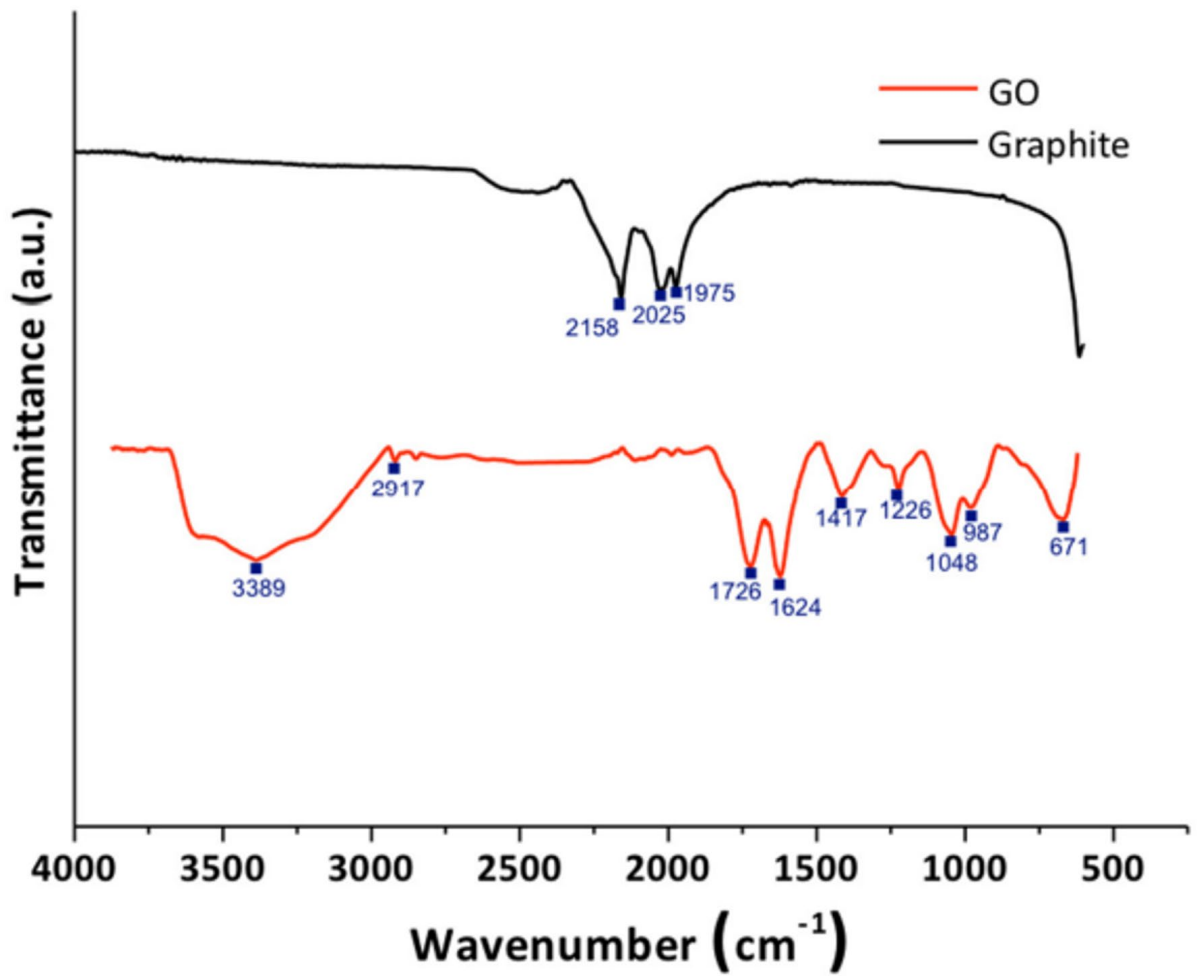
FTIR spectra of graphite and graphene oxide nanoparticles.

Figure 7 Shows the FTIR spectrum of graphite and graphene oxide. For the graphite, it exhibits few absorption signals, due to the difference in the state of charges between carbon atoms. Graphite exhibits minimal IR absorption due to its symmetric, non-polar structure^39^. It was clear that bands corresponding to oxygenated functional groups appear when an oxidation treatment was applied to the material, indicating that the oxidative process was successful. The graphene oxide spectrum showed a band at 3389 cm^− 1^, which corresponds to the stretching vibration of the hydroxyl groups (O–H). The band at 2917 cm^− 1^ corresponds to the vibration of the (C–H) bond that was in sp^3^ hybridization. The band at 1726 cm^− 1^ corresponds to the stretching vibration of the carbonyl group (C=O). In addition, the band at 1624 cm^− 1^ corresponds to the stretch mode of the sp^2^ carbon skeletal network (C=C). It was also evident, according to some results reported in the literature, that the GONPs obtained contains several oxygen functional groups, such as hydroxyl, carboxyl, and epoxy groups, which correspond to the bands presented at 1417, 1226, 1048, 987, and 671 cm^− 1^, respectively^40,41^.

#### Raman spectroscopy

**Fig. 8:**
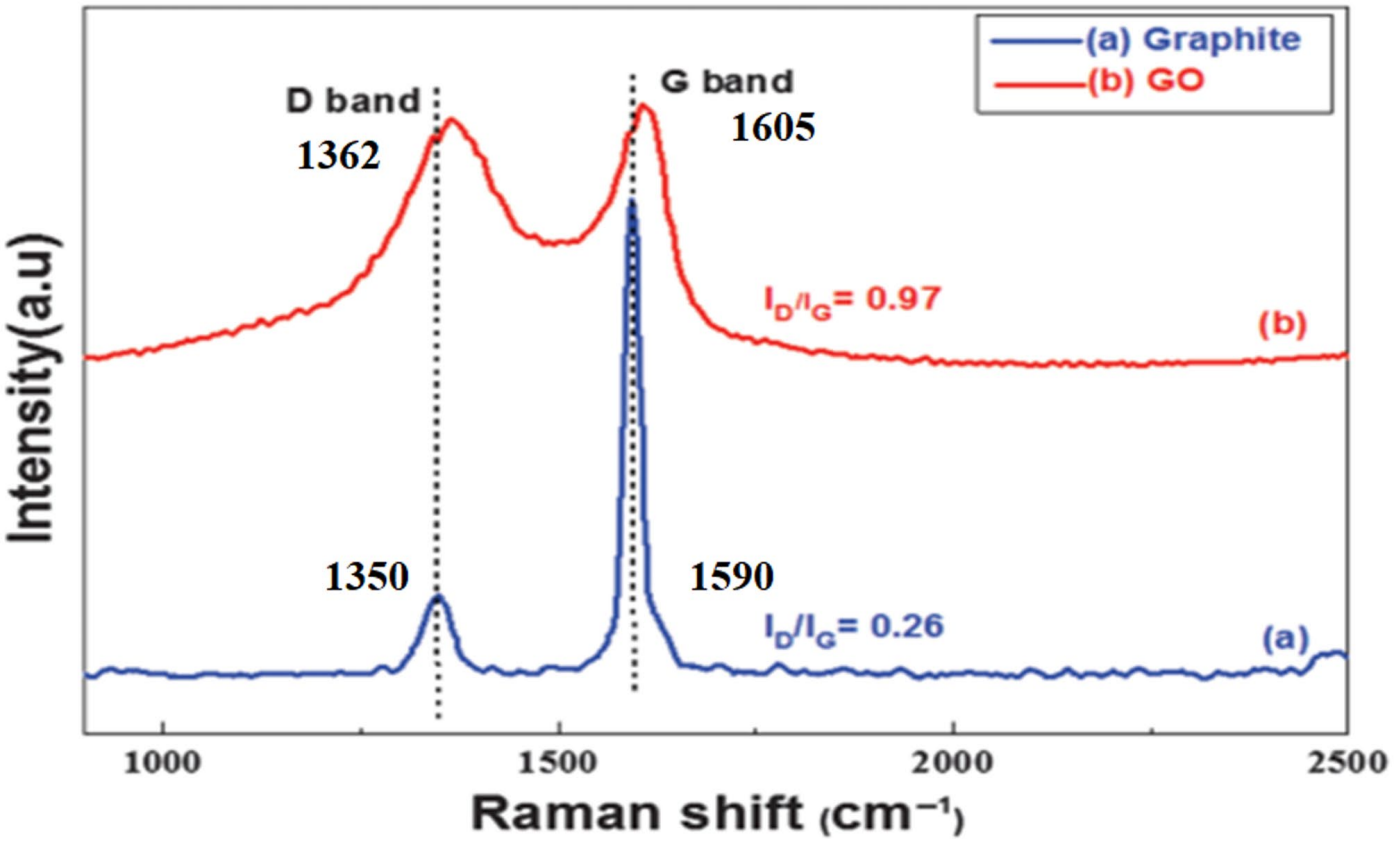
Raman spectra of (**a**) graphite powder and (**b**) GONPs.

Raman spectroscopy was based on the inelastic scattering of light, which was highly sensitive to changes in the polarizability of a molecule’s electron cloud during vibrations. Figure 8 shows the Raman spectrum of graphite and graphene oxide Nanoparticles. Graphite’s Raman spectrum showed a modest D band at 1350 cm^− 1^ because of flaws or disorder in the hexagonal structure, this peak arises from the in-plane C=C bond stretching vibration and was characteristic of the sp^2^ bonded carbon structure, and a very strong G band at 1590 cm^− 1^, This peak indicates structural disorder, such as defects or edges within the graphene lattice, and its intensity relative to the G band (I_D_/I_G_ ratio) serves as a measure of sample quality^42^. When graphite was oxidized and exfoliated to GONPs, the G and D bands underwent significant modifications. Both the D and G bands in the GONPs Raman spectra were moved to the right and widened. The appearance of the D and G bands was 1362 cm^− 1^ and 1605 cm^− 1^, respectively. During the chemical conversion process, the size and Crystallinity of the graphitic materials decreased, while the isolated graphene domain increased. The Raman spectroscopy results indicate that the GONs were formed from graphite, and they also contribute to an increased in the ratio of D band to G band intensity, or I_D_/I_G_, from graphite to GO (0.27 to 0.97) indicating a high level of cyclic disorder and the introduction of oxidized functional groups into the structure.

#### Scanning electron microscopy

**Fig. 9:**
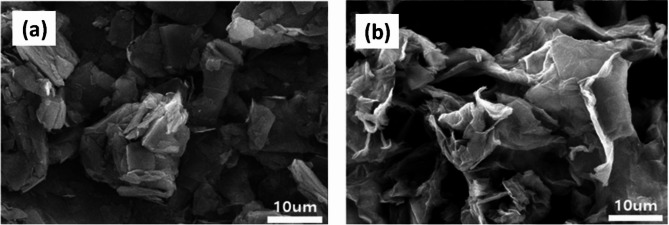
SEM image of (**a**) graphite powder (**b**) graphene oxide GONPs.

A field emission scanning electron microscope was used to study the surface morphology of graphene oxide and graphite powder. Figure 9a showed the stacked layer structure of graphite powder. Graphene oxide Nanoparticles (GONPs) shape was very different from graphite’s, and Fig. 9b showed that GONPs was made up of crumpled particles that were randomly aggregated and strongly correlated with each other through π–π contact^43,44^.GONPs were crumpled during the restacking and exfoliation process.

### Characterization of the modified and unmodified PVDF membranes

#### The surface morphology of modified and unmodified PVDF membranes at different GONPs loading

**Fig. 10:**
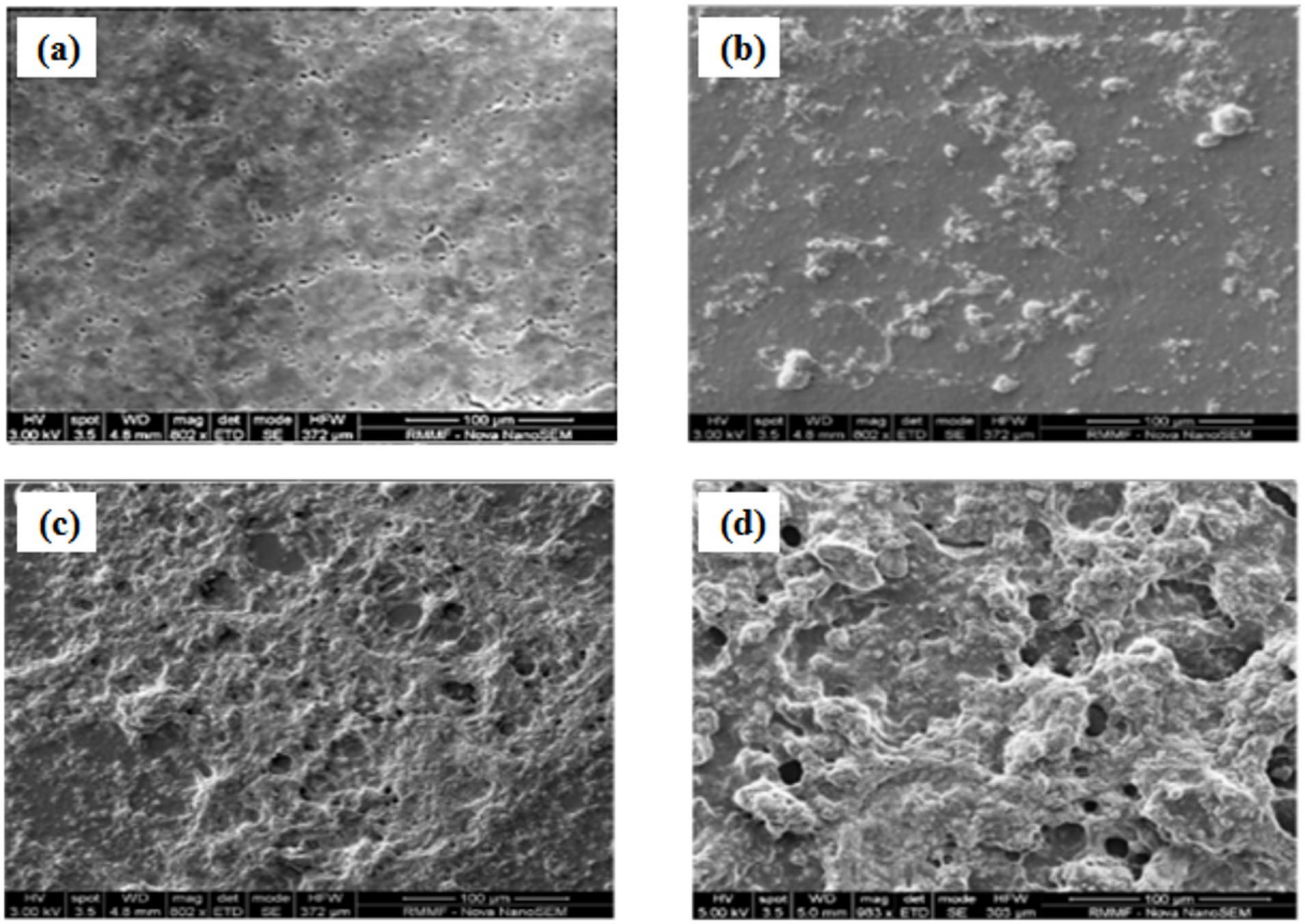
SEM images of the PVDF membranes at different GONPs loading (**a**) = 0; (**b**) = 0.1; (**c**) = 0.3; and (**d**) = 0.5 wt % GONPs.

SEM analysis was used to look at the modified and unmodified PVDF membranes’ surface morphology. The surface of the unmodified PVDF membrane shows some obvious macrospores, whereas the modified membranes showed a layer of GONPs/PVA over the substrate, as shown in Fig. 10a. At lower concentrations (i.e.0.1wt% GONPs), the distribution of GO nanoparticles was irregular; however, as GONPs loading increased, the distribution became uniform across the membrane surface, with some of the particles clumped together on the surface, as seen in the SEM images Fig. 10. When the GONPs loading was increased to 0.5 wt%, the nanoparticles might have incorporated into the membrane’s pores, reducing the membrane’s porosity. At high concentrations, aggregates can block membrane pores and restrict water flow, increasing hydraulic resistance and decreasing flux. Aggregates can protrude from the membrane surface, increasing its roughness. This uneven topography provides more sites for foulants to attach and accumulate. Polyvinyl alcohol (PVA) was a common additive used to improve nanoparticle dispersion and membrane quality. PVA’s stabilizing effect ensures that nanoparticles remain well-dispersed throughout the polymer casting solution, leading to a more homogenous and uniform distribution of nanoparticles in the final membrane matrix, and helps control the polymer’s behavior, promoting the formation of a smooth, defect-free selective layer.

#### The GO nanocomposite PVDF membrane’s surface roughness analysis

##### Surface roughness measurements

Surface roughness for all the membranes was measured using an atomic force microscope (AFM). Small part of the prepared membranes (≈ 1.5 × 1.5 cm^2^) was glued on a flat and hard piece of glass. The membrane was scanned and imaged in a scan size of 2 × 2 µm^2^. Roughness parameters were obtained and reported in terms of root mean square roughness (RMS) and mean surface roughness (Sa). Recorded results were the average of three measurements of a sample.

##### PVDF membrane’s surface roughness analysis

**Fig. 11:**
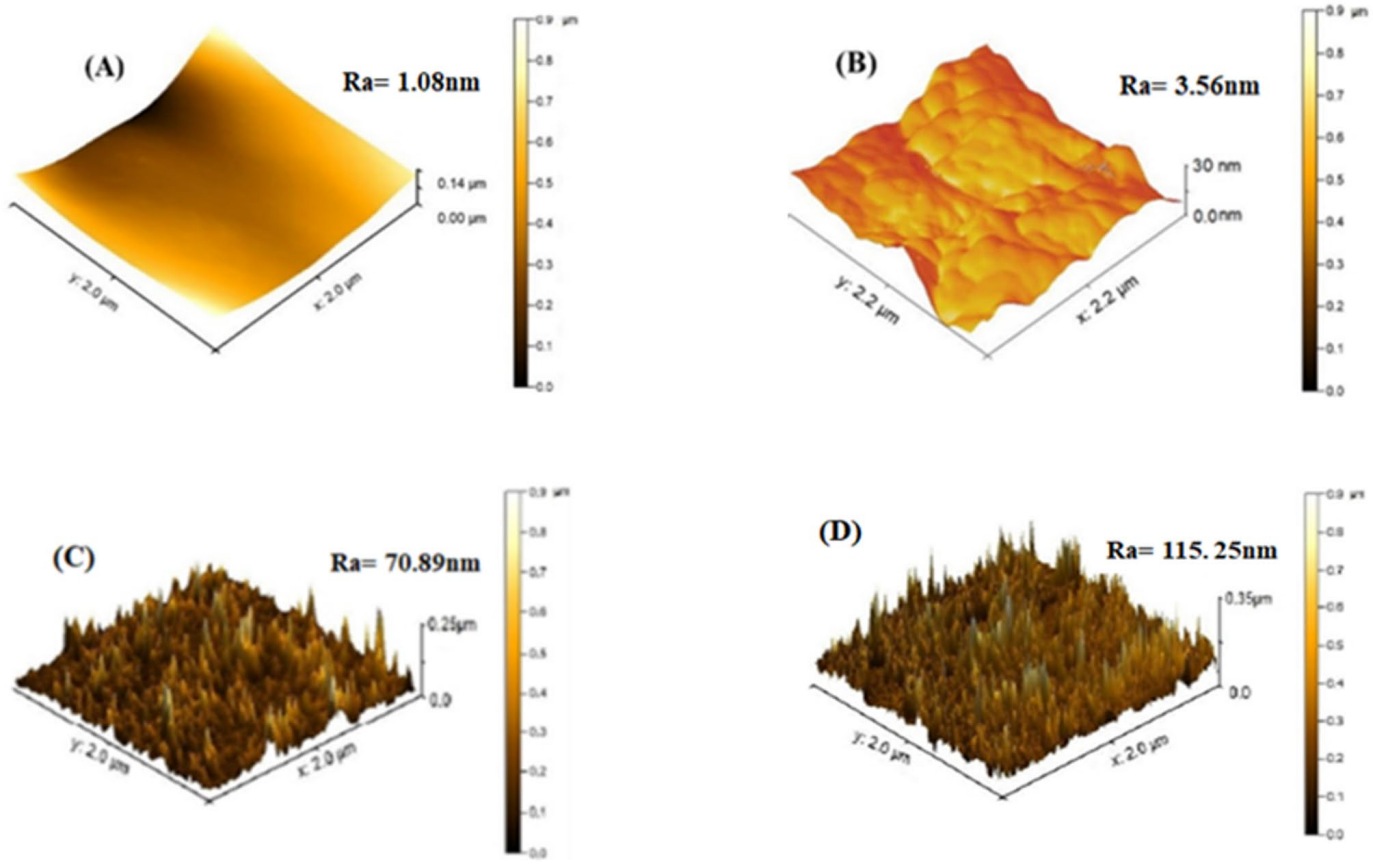
AFM images of the PVDF TFC membranes at different GONPs loading (**A**) = 0; (**B**) = 0.1; (**C**) = 0.3; and (**D**) = 0.5wt. % GONPs.

The surface topography is one of the powerful techniques for mapping the surface morphology, roughness, and adhesive properties of the nanoparticles on the PVDF membrane with and without modification. The average value of root means square (RMS) and the average roughness (Ra) of different membranes were presented in Table 1. It can be observed that the GO loading has increased the surface roughness of the modified membrane. AFM images show that the membrane surface possesses peak and valley like structures (the brightest parts in each AFM images represent the peak, whereas the darkest parts represent the valleys). The increasing surface roughness with increasing GO loading confirms the adhesion of GO nanoparticles on the TFC membrane’s surface. The increase in GO loading has also increased the peak and valley, which could help to enhance the removal of heavy metal ions. Shown in Fig. 11.

**Table 1:** Surface roughness parameters at different GO loading on the PVDF membranes obtained from AFM images.

Membrane	Root mean square roughness (RMS) (nm)	Mean roughness (Sa) (nm)
Pure PVDF	1.35	1.08
PVDF/GO(0.1wt%)	4.46	3.56
PVDF/GO(0.3wt%)	88.83	70.89
PVDF/GO(0.5wt%)	144.42	115.25

Ra = Sa = RMS × 0.798 (nm).

#### The contact angle of the membrane

Contact angle measurement is suitable technique to study and evaluate the water/PVDF interface properties i.e. hydrophobicity. The water droplet would spread out over the PVDF surface forming a contact angle that varies according to the water/surface interactions. The geometry of the water drops and interfacial contact can be visually observed and determined the angle between the tangent of the drop boundary and the drop baseline.

**Fig. 12:**
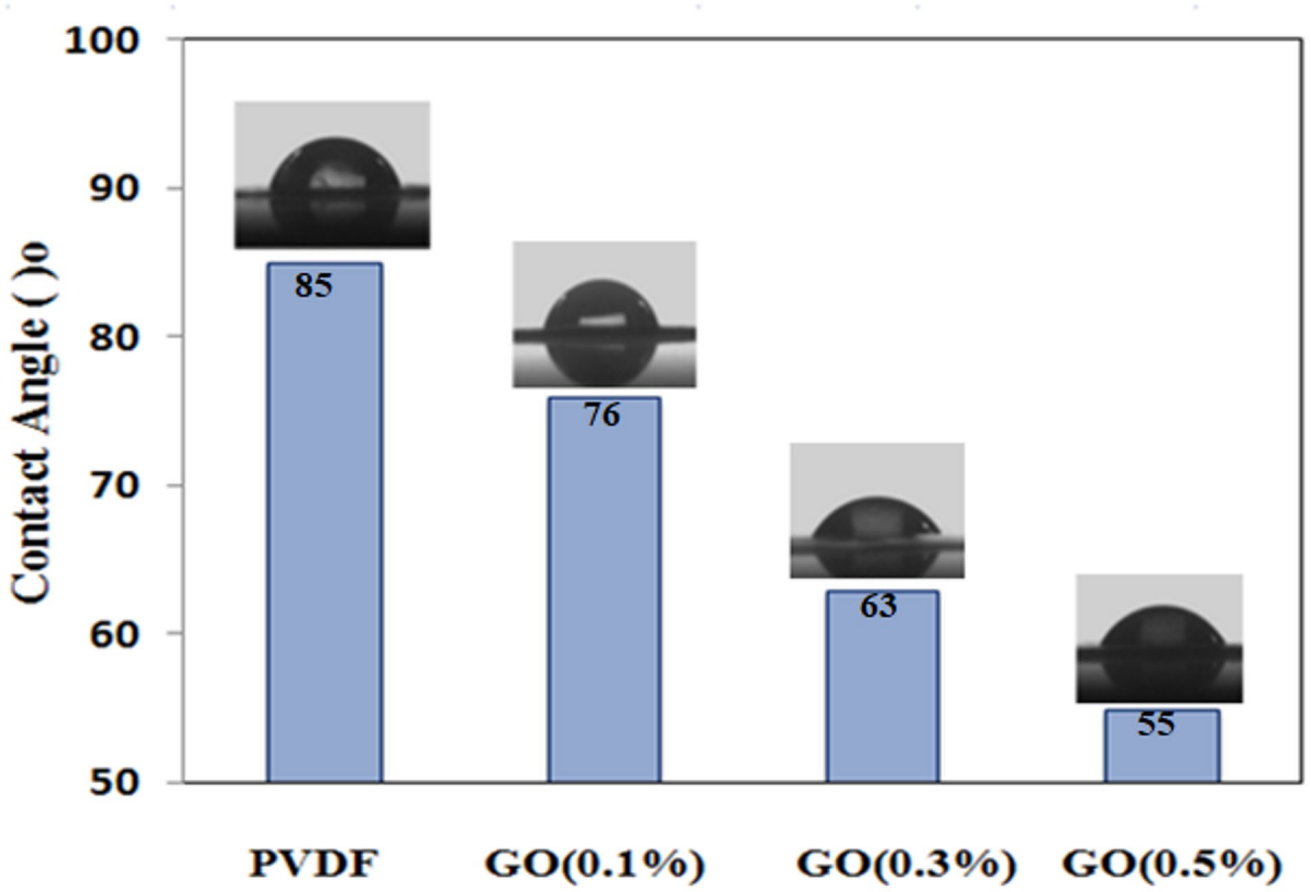
Impact of GONPs concentration on the water’s contact angle with the PVDF membranes.

The wettability of membrane was a major parameter when evaluating the water permeability of a membrane. It’s commonly believed that the degree of hydrophilicity increases water permeability^45^. The modified PVDF membranes’ and the unmodified membranes’ contact angles were displayed in Fig. 12. the contact angle of PVDF membrane decreased as the GONPs loading increased. Unmodified membranes showed a contact angle of 85° which was decreased with increasing GONPs % tell reached to 55° with 0.5 wt% GONPs. This increase in hydrophilicity relating to present of both GONPs and PVA. Both materials contain many hydrophilic groups such as hydroxyl groups were formed on the membrane surface. Besides, from the above FT/IR results, GO contains carboxylic groups which would also contribute in hydrophilicity. It can conclude that the surface modification of PVDF with PVA/GONPs enhanced the contact between water and the membrane surface^46^.

#### Membrane surface charge

The surface charge has a major effect on the removal efficiency of charged ions.

**Fig. 13:**
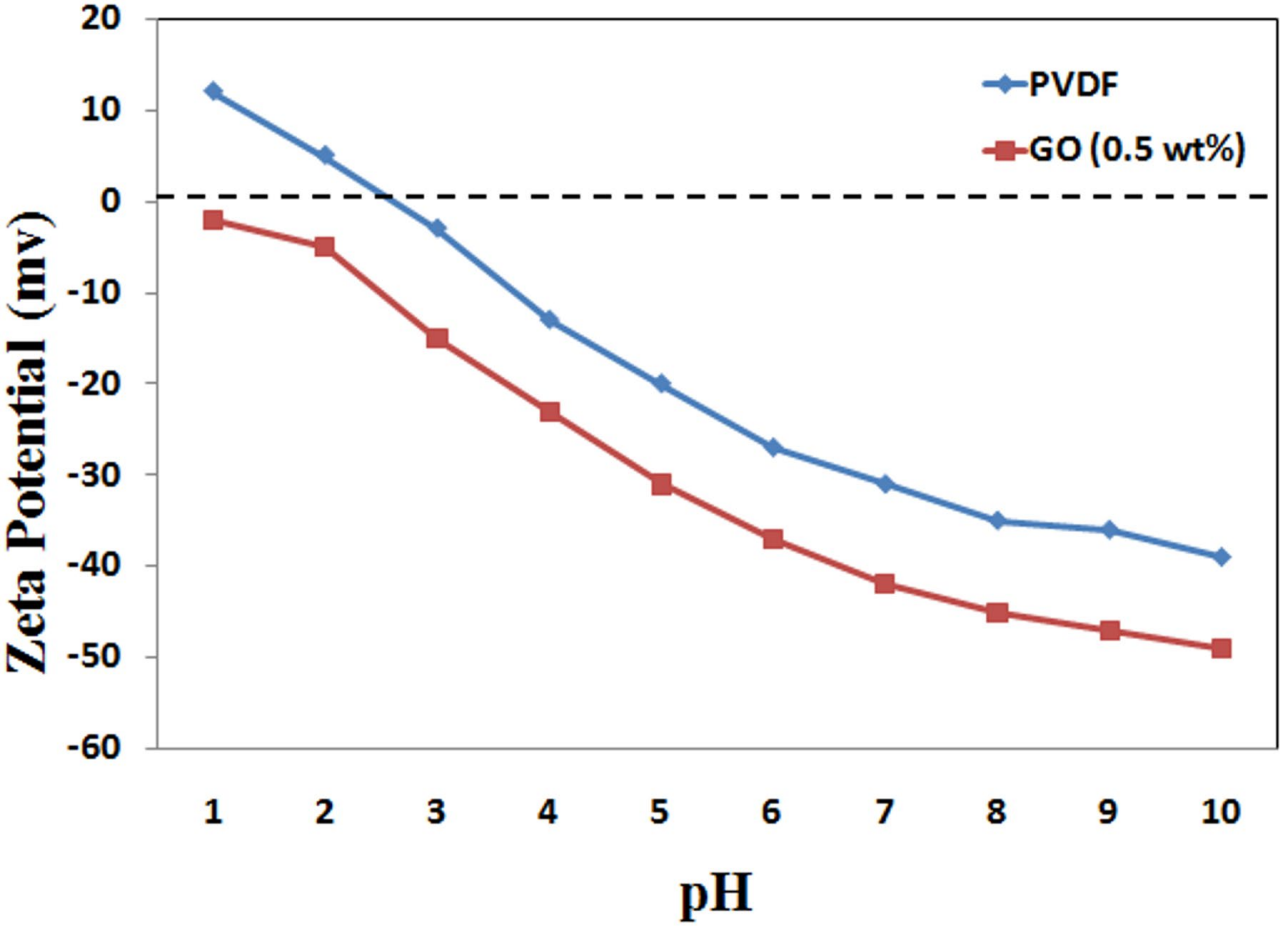
Surface charge of the plain and modified PVDF membranes at GONPs (0.5wt %).

The surface charge can be evaluated via zeta potential measurements, as shown in Fig. 13. the zeta potential (ζ) varied with the change of solution pH that would reflecting on protonating and deprotonating of the membrane’s functional group. The stability of the ions in the suspension was greatly impacted by both positive and negative values. This is explained by ion segregation, which occurs when ions with the same electric charge repel one another^47^. In acidic environments, GONPs show a negative zeta potential that increases with pH. The PVDF membrane coated with GONPs/PVA has a negative charge throughout the pH range^48^. This negative charge increase the performance of the modified PVDF membrane in the removal of heavy metals through the electrostatic interaction between the negatively charged GONPs surface and the positively charged heavy metal ions.

#### The mechanical properties of the modified and unmodified PVDF membranes

One of the main advantages of the PVDF membrane was its high mechanical strength; therefore it would be convenient to evaluate the impact of any modification on these properties. Any modification of the membrane surface would reflect in its mechanical properties. Stress- strain evaluation was one of the most suitable measurements to examine the mechanical properties, mainly tensile strength, of polymers.

**Fig. 14:**
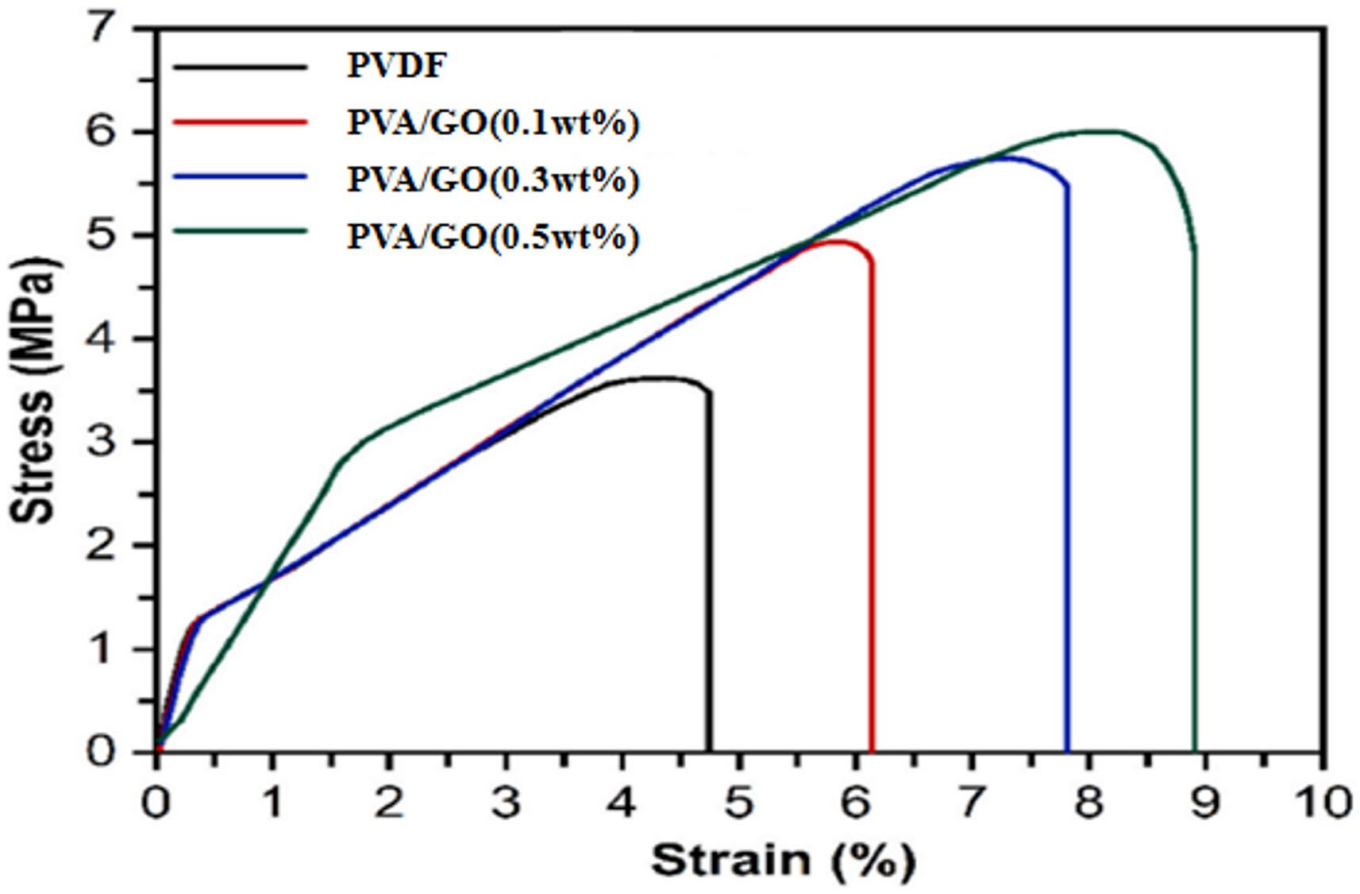
Stress–strain curves of the plain and modified PVDF membranes at different GONPs loading.

The tensile strength of a membrane was usually measured by a tensile tester registering tensile stress versus strain (i.e., extension per original length) until failing or breaking. The mechanical test results for the unmodified and modified PVDF membranes were shown in Fig. 14 and Table 2.

**Table 2:** Mechanical characteristics of both the modified and unmodified PVDF membranes.

Membrane	Tensile strength (Mpa)	Breaking elongation (%)
PVDF	3.58	4.65
GO (0.1 wt%)	4.97	6.23
GO (0.3 wt%)	5.67	7.78
GO (0.5 wt%)	6.15	8.79

**Fig. 15:**
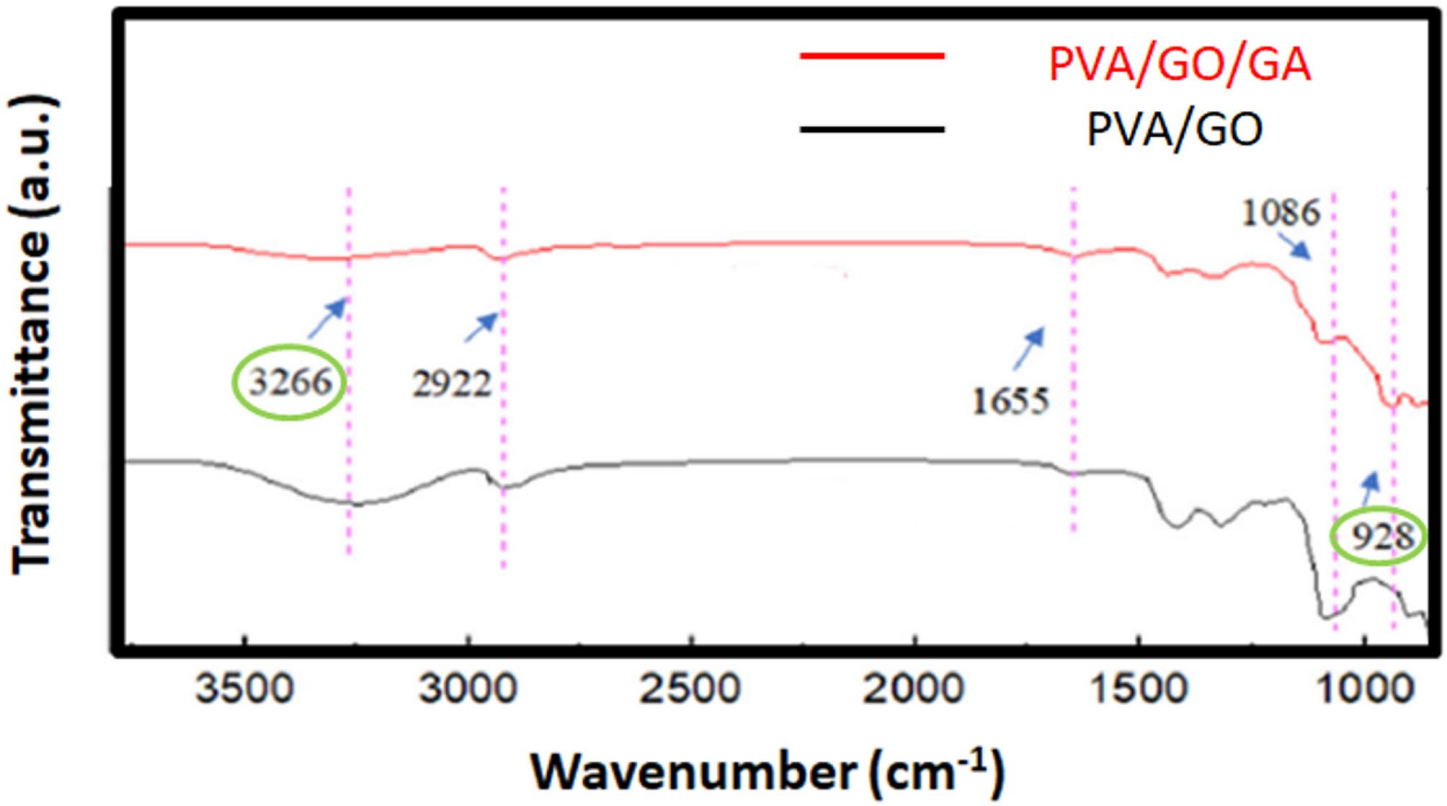
FTIR spectra of PVA/GONPs and PVA/GONPs without GA.

According to the analysis’s findings, the modified membranes’ tensile strength rose proportionately from 3.58 to 6.15 MPa when the PVA/GONPs loading increased by 0.1, 0.3, and 0.5 wt%. Most of polymeric water filter membranes have tensile strengths ranging from 3 to 15 MPa^49^. It was determined that the tensile strength increases after the crosslinking with GA .FTIR techniques revealed changes in the spectra before and after GA treatment Fig. 15. Comparing the spectrum of PVA/GONPs and PVA/GONPs without GA, it could be seen that after crosslinking, the peak of O–H situated at 3266 cm^− 1^ decreased obviously while a strong absorption peak which corresponds to the stretching vibration peak of C–O–C appeared at 928 cm^− 1^. This indicated that after GA crosslinking, the –OH on both GONPs and PVA could react with the aldehyde on glutaraldehyde simultaneously to form ether bonds. These hydroxyl aldehyde reactions can not only form a more stable crosslinking structure, but also fixed GONPs on the surface of PVDF membrane to prevent GONPs from displacement and falling off^50^. The influence of a higher PVA/GONPs concentration was associated with an increase in strain since the presence of extra hydrogen bonds and ether bonds between the PVA/GO nanocomposites enhanced the modified PVDF membrane’s resistance to elongation.

### Performance and removal efficacy of the membrane

#### Effect of solution pH on the removal of heavy metal ions

**Fig. 16:**
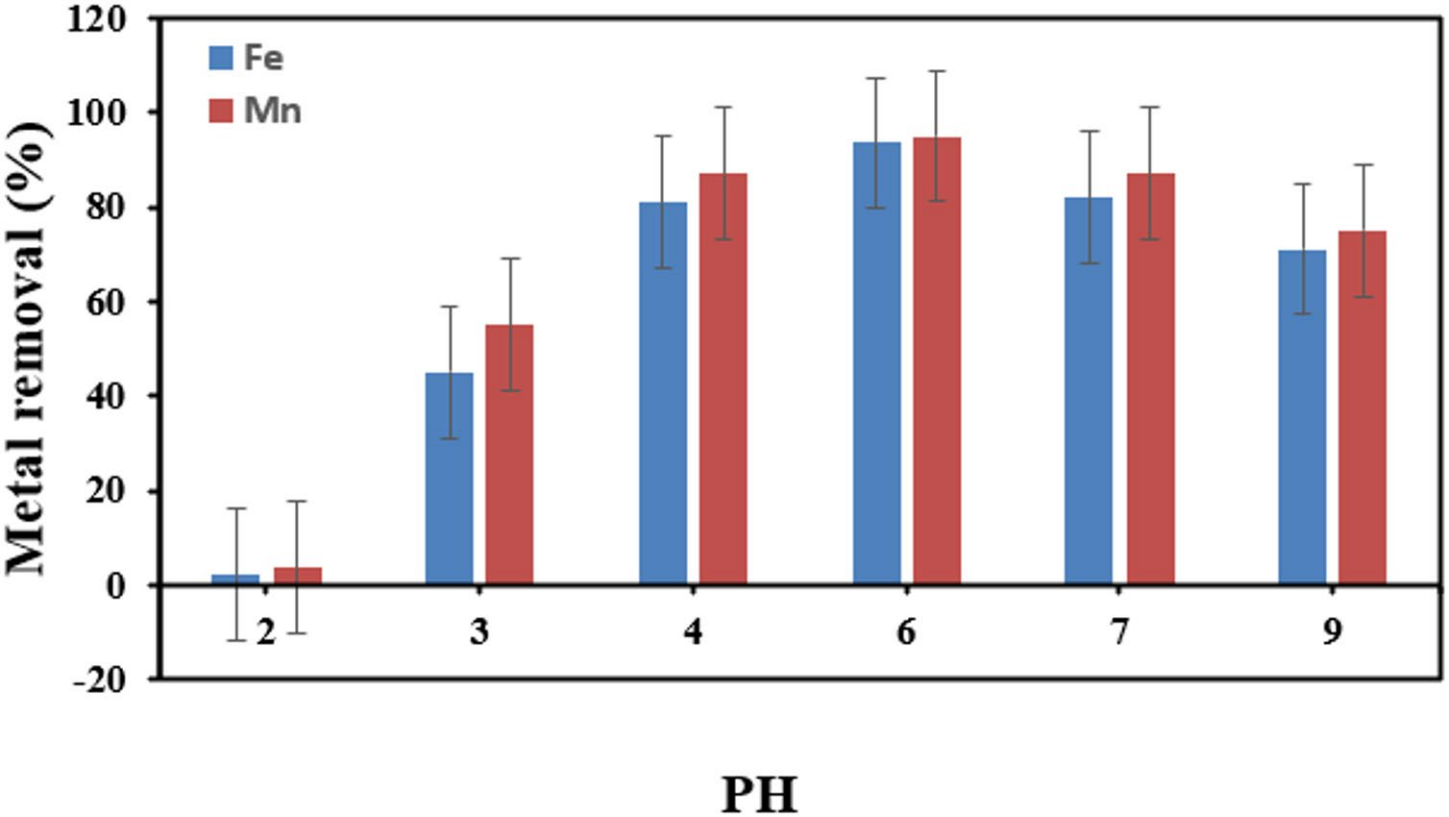
Impact of pH on GONPs/PVDF membrane’s ability to remove heavy metals.

**Fig. 17:**
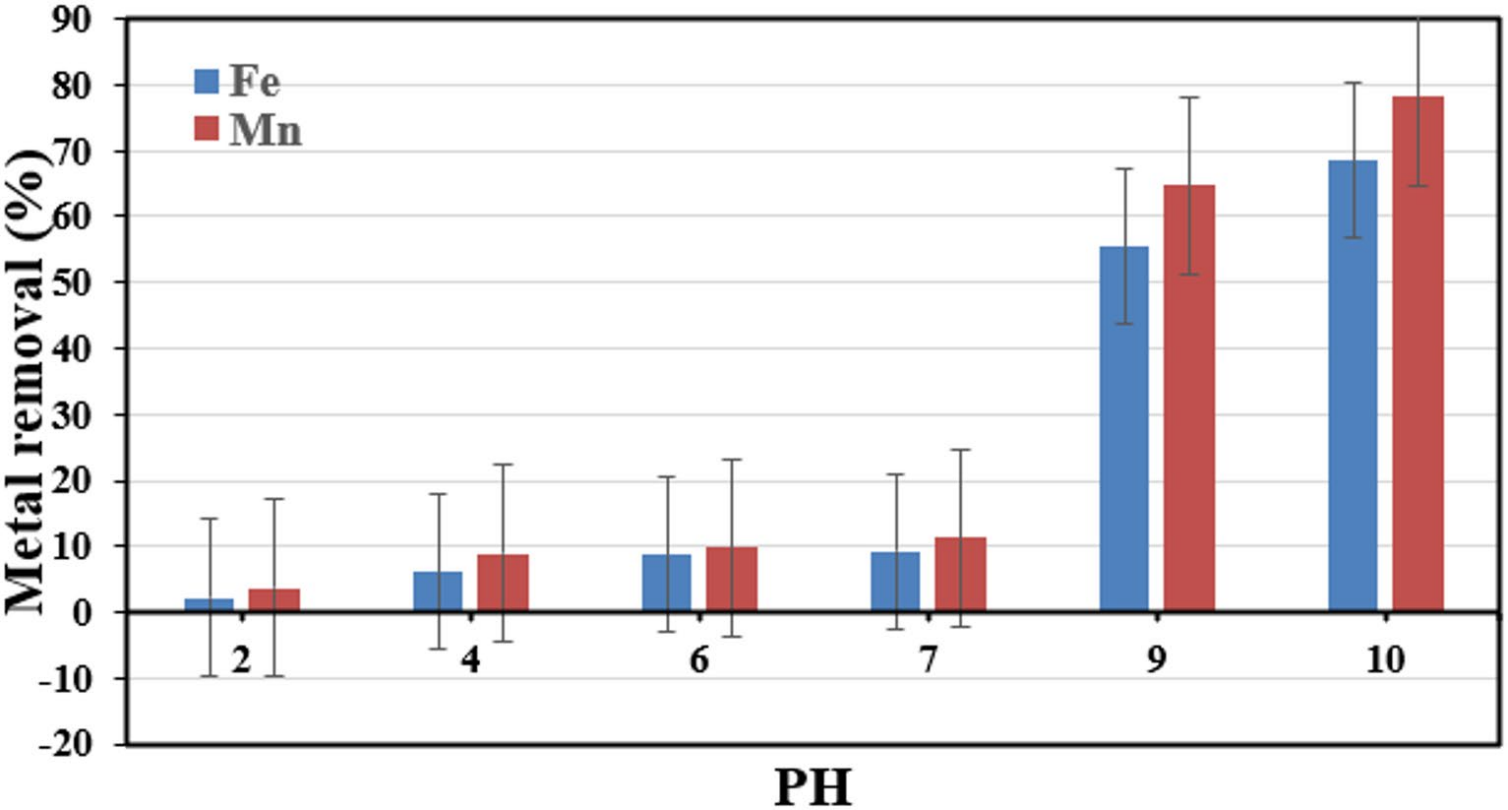
Effect of pH on the removal of heavy metals without membrane.

The electrostatic interaction between the metal ions in solution and the functional groups on the membrane surface was influenced by the initial pH value of the solution, making it a crucial parameter for metal removal^51^. For both metals, batch tests were conducted at various pH values (ranging from 2 to 9) in order to investigate the impact of pH on the Fe^2+^ and Mn^2+^ ions removal on the modified PVDF membrane. Figure 16 Demonstrates that the highest percentage of Fe^2+^ and Mn^2+^ ions removal on the modified PVDF membrane was recorded at pH values between 4 and 7, where the maximum removal efficiency of Fe^2+^ was 93.5% and for Mn^2+^ was 95% at pH 6. This may be explained by the hydrogen ions’ competition with the stated metal ions for exchangeable cations on the membrane’s surface at lower pH values. The deprotonation of carboxylic groups on the membrane surface, on the other hand, takes place in the pH range of 4.0 to 6.0, which facilitates improved metal removal^52^. Figure 17 Demonstrates that the highest percentage of Fe^2+^ and Mn^2+^ ions removal without using membrane at higher pH values. This may be explained by the precipitation of Fe^2+^ and Mn^2+^ ions as hydroxides.

At high pH levels, both Fe^2+^ and Mn^2+^ will precipitate as their respective insoluble hydroxides, Fe (OH)_2_ and Mn (OH)_2_, because increasing pH increases the concentration of hydroxide ions (OH⁻) in the solution. The solubility product constant (K_sp_) for these compounds dictates the equilibrium between the solid hydroxide and the dissolved metal ions. The solubility product constant (K_sp_) quantifies the solubility of these precipitates. For Fe (OH)_2_, the K_sp_ expression was [Fe^2+^][OH^−^]^2^, and for Mn(OH)_2_, it’s [Mn^2+^][OH^−^]^2^.

As the pH increases, [OH^−^] increases, which drives the precipitation reaction to the right and decreases the concentration of dissolved metal ions to maintain the K_sp_ equilibrium.

#### Water flux of the unmodified and modified PVDF membranes

**Fig. 18:**
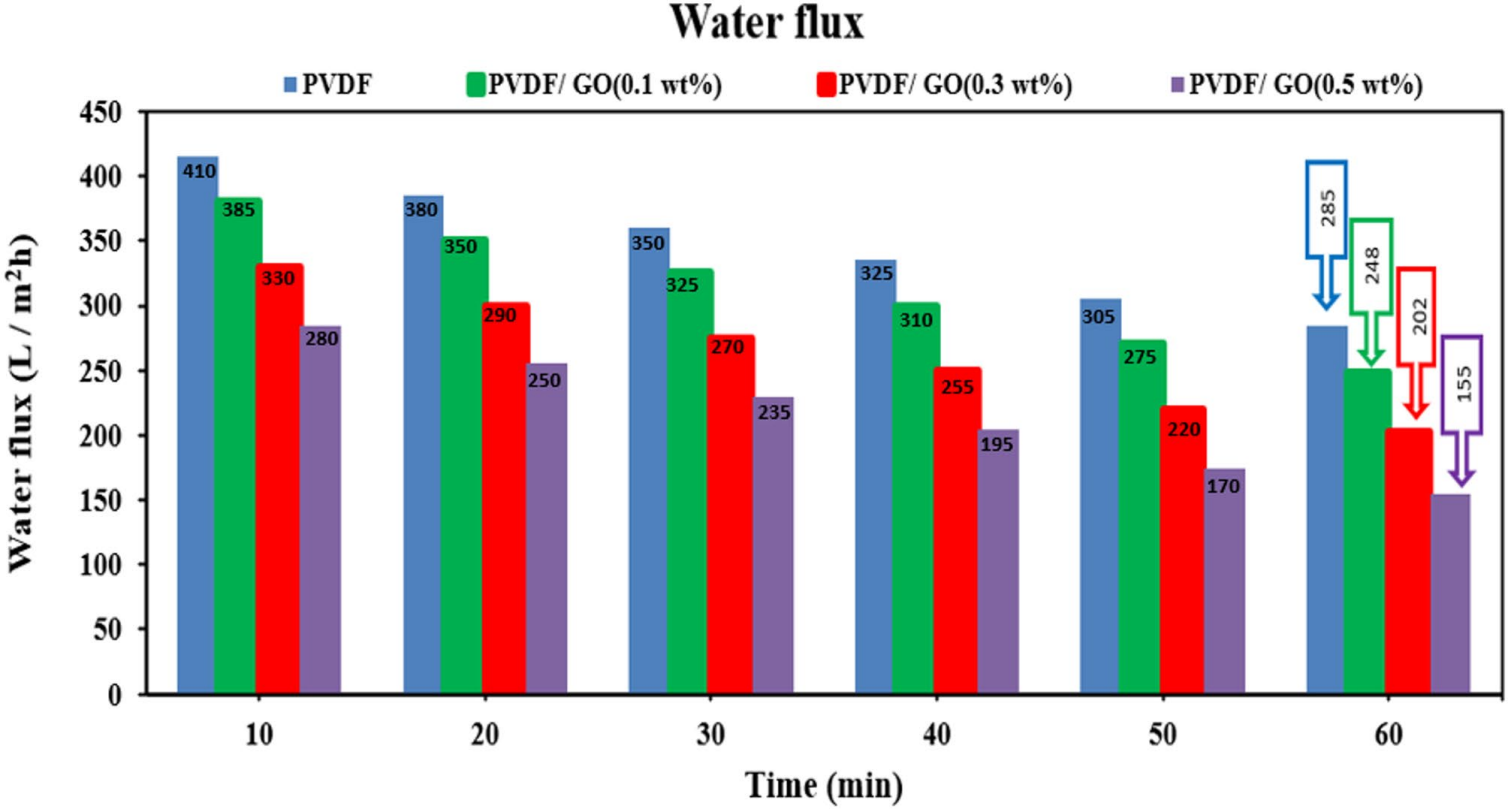
Water flux of the plain and modified PVDF membranes at different GONPs loading.

Water flux were measured at an operating pressure of 3.0 bars, the flow rate was maintained at 18 L/h. while the membrane with an active filtration area of 42.0 cm^2^ and 100 ppm solutions of Fe^2+^ and Mn^2+^ metal ions at pH 6. Water flux fluctuations of membranes coated with GONPs (0.1, 0.3, and 0.5 wt%) nanoparticles were depicted in Fig. 18. A high initial flux may be due to a large osmotic pressure difference and low resistance, which allows for a rapid net movement of water. A decline in flux over time was a common phenomenon. The permeate flux was reduced from (285 L/m^2^ h) for pure PVDF membrane to (248 L/m^2^ h) for modified PVDF/GO(0.1wt%) and an increase in GO nanoparticles loading up to GO (0.5wt%) the flux decreased to (155 L/m^2^ h). As the GO loading increases, the membrane structure becomes denser and thicker. This reduces the number and size of pores available for water to pass through, resulting in a significant decrease in water flux, while enhancing salt rejection due to the tighter Nano channel structure. This trade-off occurs because GO, in high concentrations, forms a more compact and denser matrix, restricting water passage but creating a more effective barrier against ion^53^. The above SEM morphology analysis demonstrated that an increase in GO nanoparticles loading improves the dispersions and decreases the size of the pores by forming a thin layer of GONPs/PVA on the PVDF membrane’s surface.

#### Removal of heavy metal ions by unmodified and modified PVDF membranes

**Fig. 19:**
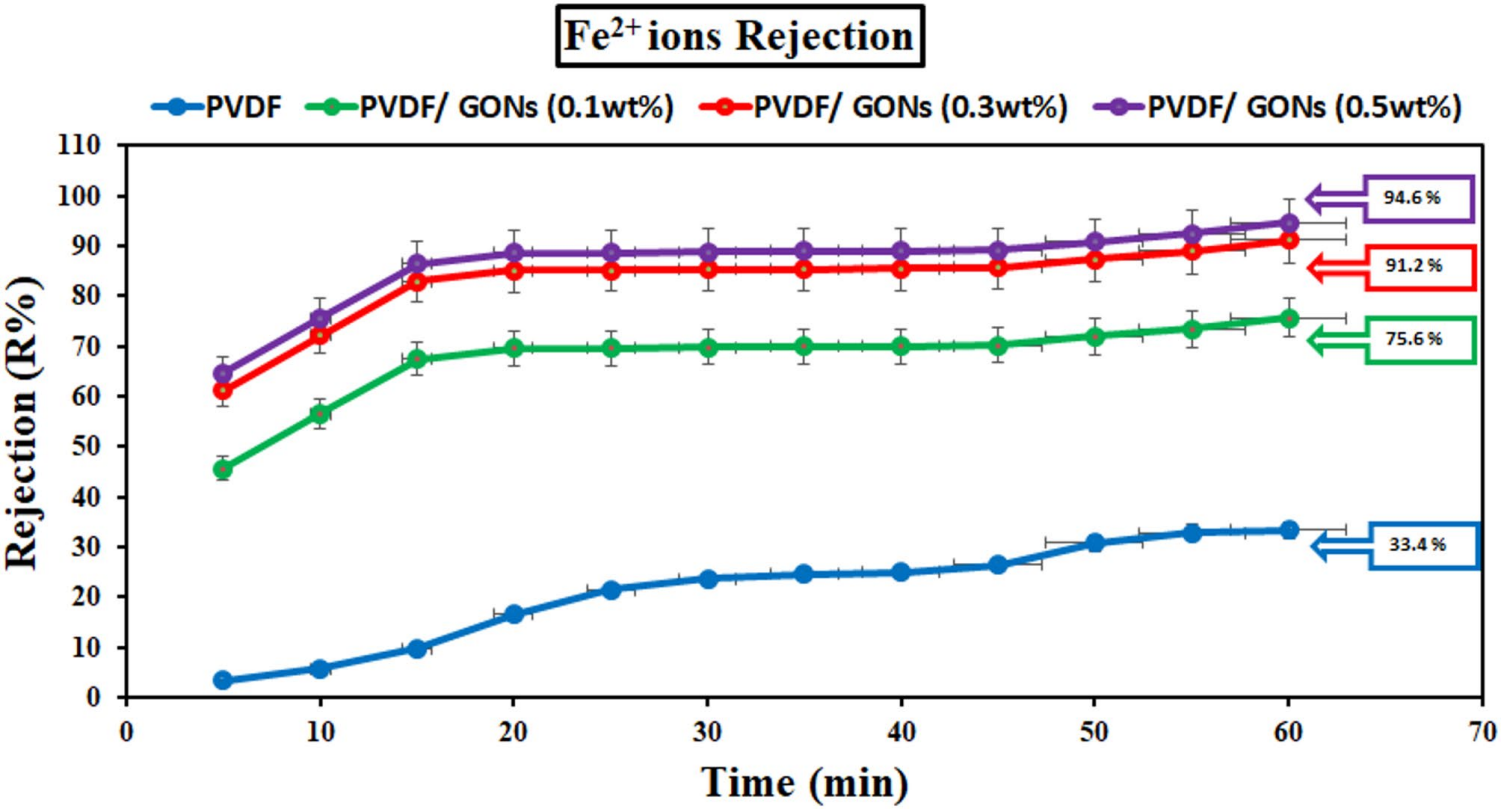
Effect of contact time on the removal ofFe^2+^ ions at pH 6 and at different GONPs loading.

**Fig. 20:**
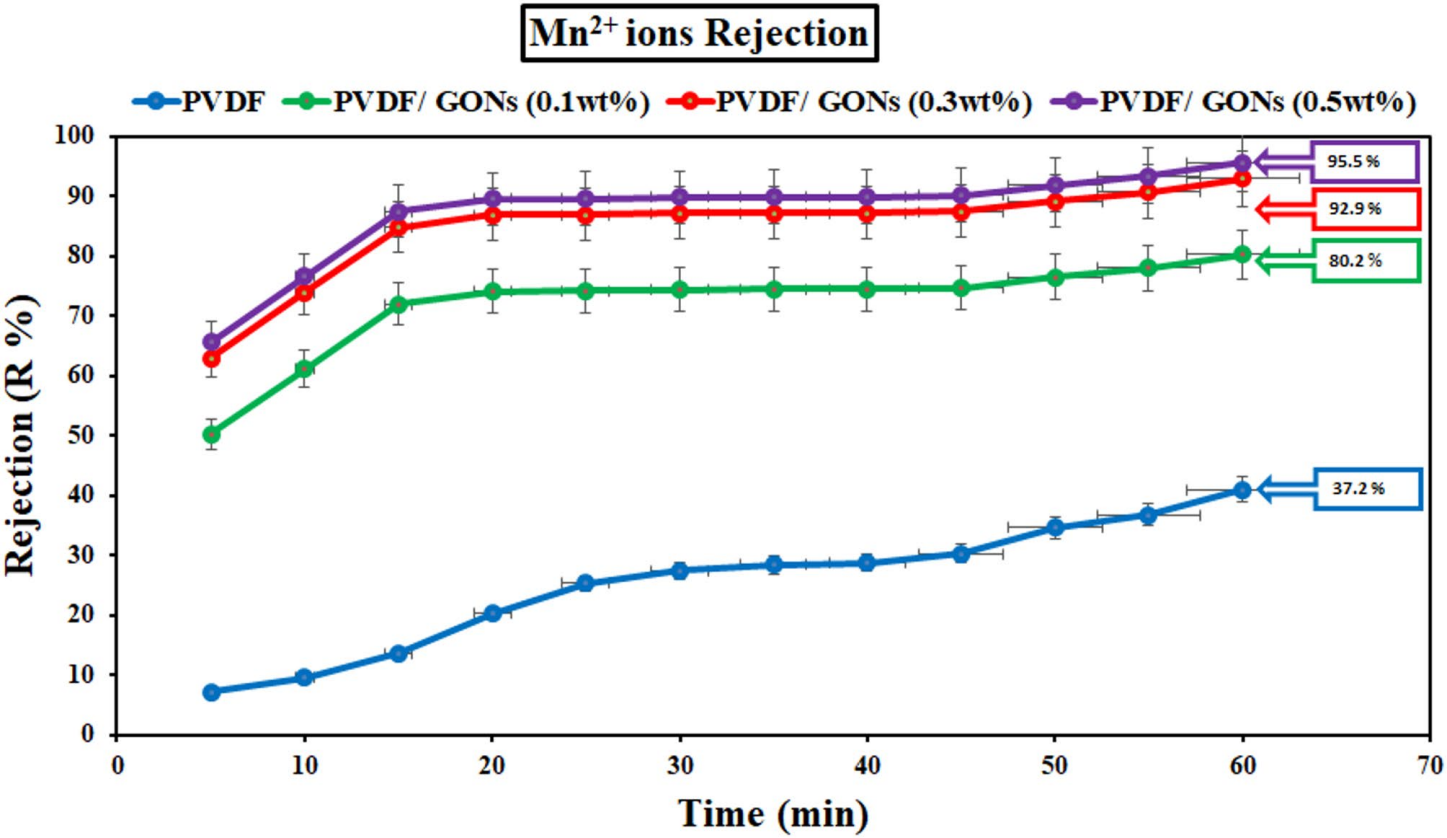
Effect of contact time on the removal ofMn^2+^ ions at pH 6 and at different GONPs loading.

Contact time was a crucial parameter influencing the efficiency of heavy metal ion removal from contaminated water, particularly for ions such as Fe^2+^ and Mn^2+^. The results presented in Figs. 19 and 20 highlight the significant improvement in removal efficiency when modified PVDF membranes were used. Specifically, the removal efficiency of Fe^2+^ ions increased dramatically from 33.4% for unmodified PVDF membranes to 94.6% for the modified PVDF membranes, while Mn^2+^ removal efficiency increased from 37.2% to 95.5% under similar conditions.

The removal of both Fe^2+^ and Mn^2+^ ions follows a rapid trend during the initial 60 min of contact, as demonstrated by the figures. The significant enhancement in removal efficiency can be attributed to the unique properties of graphene oxide (GO) nanoparticles, which have been incorporated into the membrane. GO nanoparticles were known for their superior conductivity, abundant surface functionality, and relatively large surface area, all of which play a pivotal role in facilitating the adsorption of metal ions. This rapid removal during the first 60 min was consistent with previous studies that have highlighted the enhanced adsorption capacity of GO-modified membranes due to their increased surface interactions and electrostatic attraction to metal ions^54,55^.

However, as illustrated in Fig. 19, after the initial adsorption phase, the removal rate begins to slow down significantly. This observation suggests a possible exhaustion of active sites available for adsorption, leading to a plateau in removal efficiency. The reduction in the rate of ion removal can be attributed to the “fatigue” of the adsorption sites, as the surface of the modified PVDF membrane becomes saturated with metal ions. This behavior was commonly observed in adsorption processes and was in agreement with studies that show a decrease in removal efficiency as the adsorption sites become filled over time^56^.

The sharp decline in the removal efficiency beyond 60 min can also be linked to the competition between metal ions for limited adsorption sites, as well as potential desorption effects where adsorbed ions may re-enter the solution. This phenomenon has been well-documented in the literature^57^, where saturation of adsorptive surfaces leads to reduced overall efficiency despite extended contact times.

**Fig. 21:**
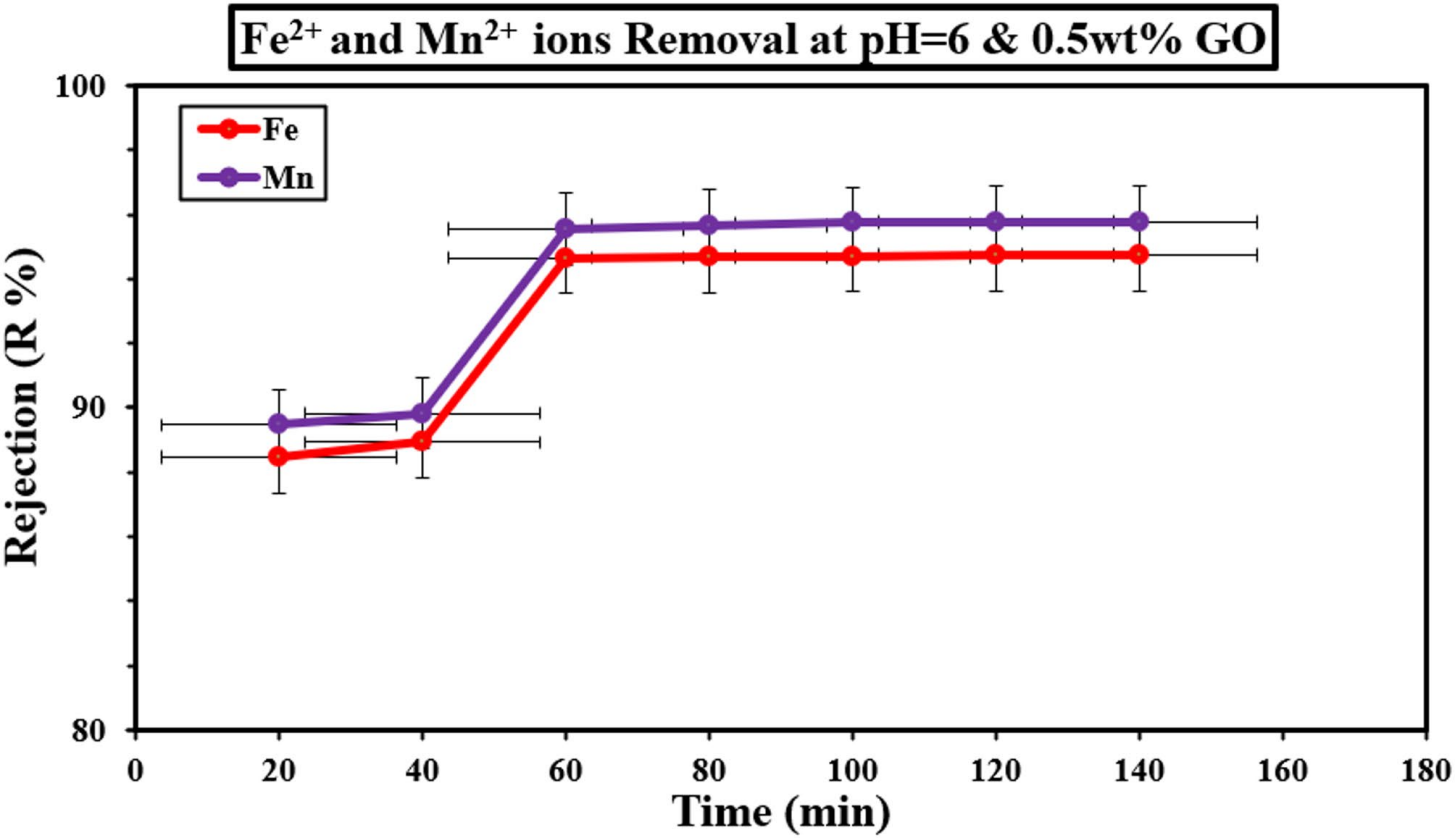
Effect of contact time on the removal of Fe^2+^ and Mn^2+^ ions at pH 6 and 0,5wt% GONPs.

In conclusion, the data from Figs. 19, 20 and 21 provide a clear understanding of the temporal dynamics involved in the removal of Fe^2+^ and Mn^2+^ ions using modified PVDF membranes. The enhanced removal efficiency observed in the early stages of contact time can be attributed to the unique properties of GO nanoparticles, while the subsequent decrease in removal efficiency can be explained by the exhaustion of adsorption sites and potential desorption phenomena.

#### Mechanism underlying metal ions removal on PVA/GONPs-PVDF membranes

**Fig. 22:**
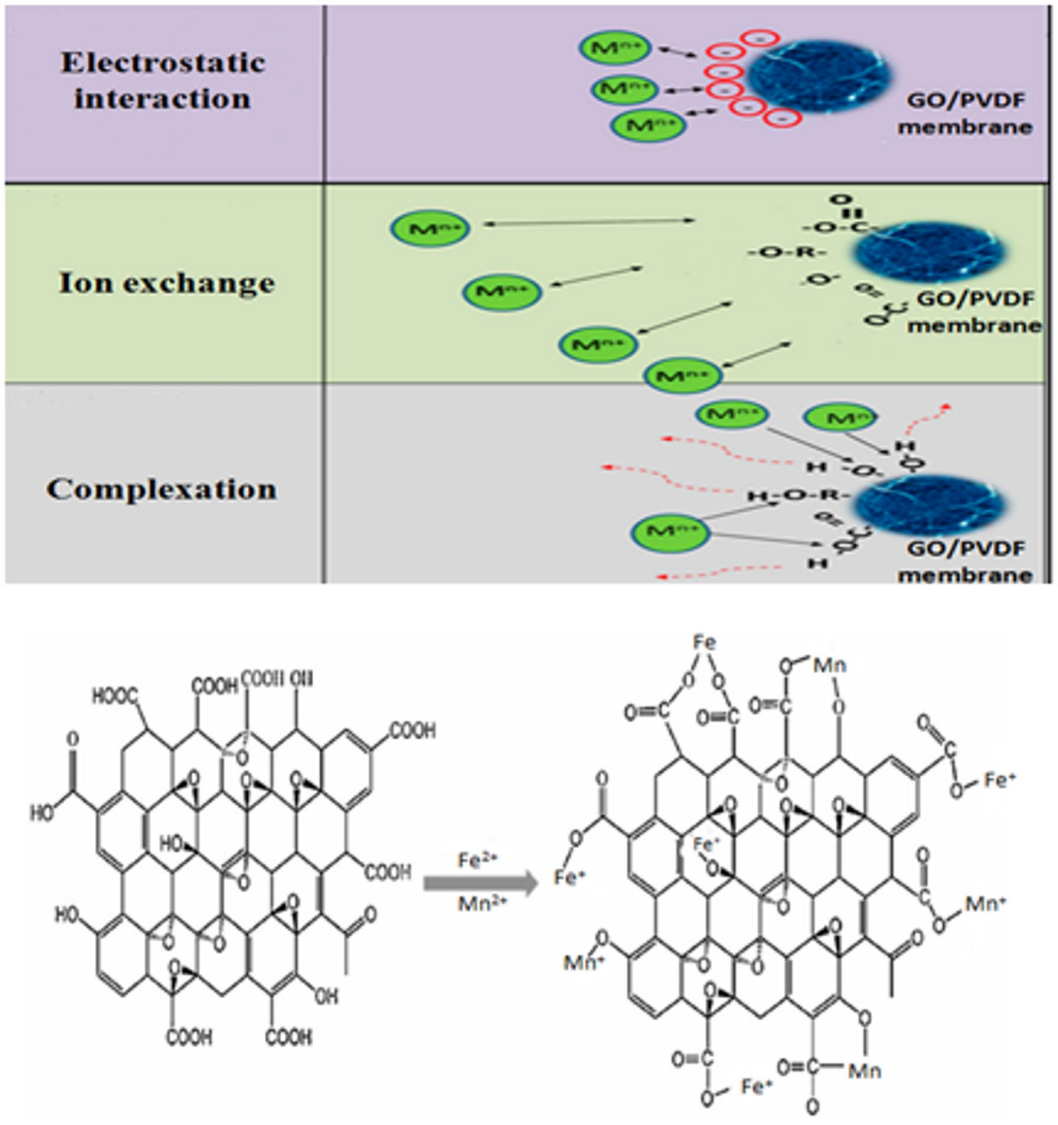
Mechanisms of metal ions attracted on the surface of GONPs/ PVDF membrane.

In order to understand how metal ions bind to the membrane, it was essential to identify the functional groups responsible for metal ions binding. Electrostatic attraction, ion exchange, and surface complexation were the three main forms of interactions that take place when metal ions were attracted on the surface of graphene oxide nanoparticles (GONPs)^51^. Figure 22 illustrates the three main possible mechanisms of metal ions attracted on the surface of graphene oxide.

One of the main factors in the adsorption process was thought to be the electrostatic interaction between the negatively charged GONPs surface and the positively charged heavy metal ions. Some heavy metal ions have been found to be adsorbed by electrostatic interaction^58,59^. The second possible contributing factor was the ion exchange reaction between heavy metal ions and protons of oxygen that contain functional groups like –COOH or –OH. It has been observed that the epoxide and hydroxyl groups were located at the basal planes of GO Nanoparticles whereas the carbonyl and carboxyl groups were most likely located close to the edges of GO Nanoparticles^60^. The process of adsorption resulted in the release of the proton of –COOH or –OH into the solution^61,62^.

The bonding of Fe^2+^ and Mn^2+^ ions was facilitated by the two primary functional groups, –COOH and –OH. The complexation processes occurring on the surface of GONPs constitute the third component. A significant factor in the adsorption of Fe^2+^ and Mn^2+^ ions on the surface-active sites of GONPs was the surface complexation between the heavy metal ions and the oxygen-containing functional groups^63,64^. As a bridge between different GO nanoparticles, the oxygen-rich functional groups at the edges of GO nanoparticles actively participate in the complexation of Fe^2+^ and Mn^2+^ ions by simultaneously joining the –COOH and –OH groups at the margins^65^.

In contrast to other oxygenated functional groups, Fe^2+^ and Mn^2+^ ions prefer to bond with –COOH during their adsorption on the GONPs surface, as demonstrated by sufficient thermodynamic data and density functional theory calculations^66,67^.

The negatively charged functional groups of graphene oxide Nanoparticles (GONPs) establish an electrostatic contact with the positively charged heavy metal ions as pollutants, and the presence of various functional groups on the edges and basal plane of GONPs enhances its potential as an adsorbent^68–70^.

#### Stability of PVA/GONPs coated onto the PVDF membrane surface during filtration process

The stability of the PVA/GONPs over the surface of the PVDF membrane plays a crucial role in the overall performance of the membrane. To evaluate the long-term stability, the modified PVDF membrane was tested over five successive filtration cycles, each cycle consisting of a 1 h stirring period in a heavy metal ion solution, followed by washing with deionized water and drying at room temperature before reuse. The membranes were repeatedly employed for the removal of Fe^2+^ and Mn^2+^ ions under the same operating conditions.

**Fig. 23:**
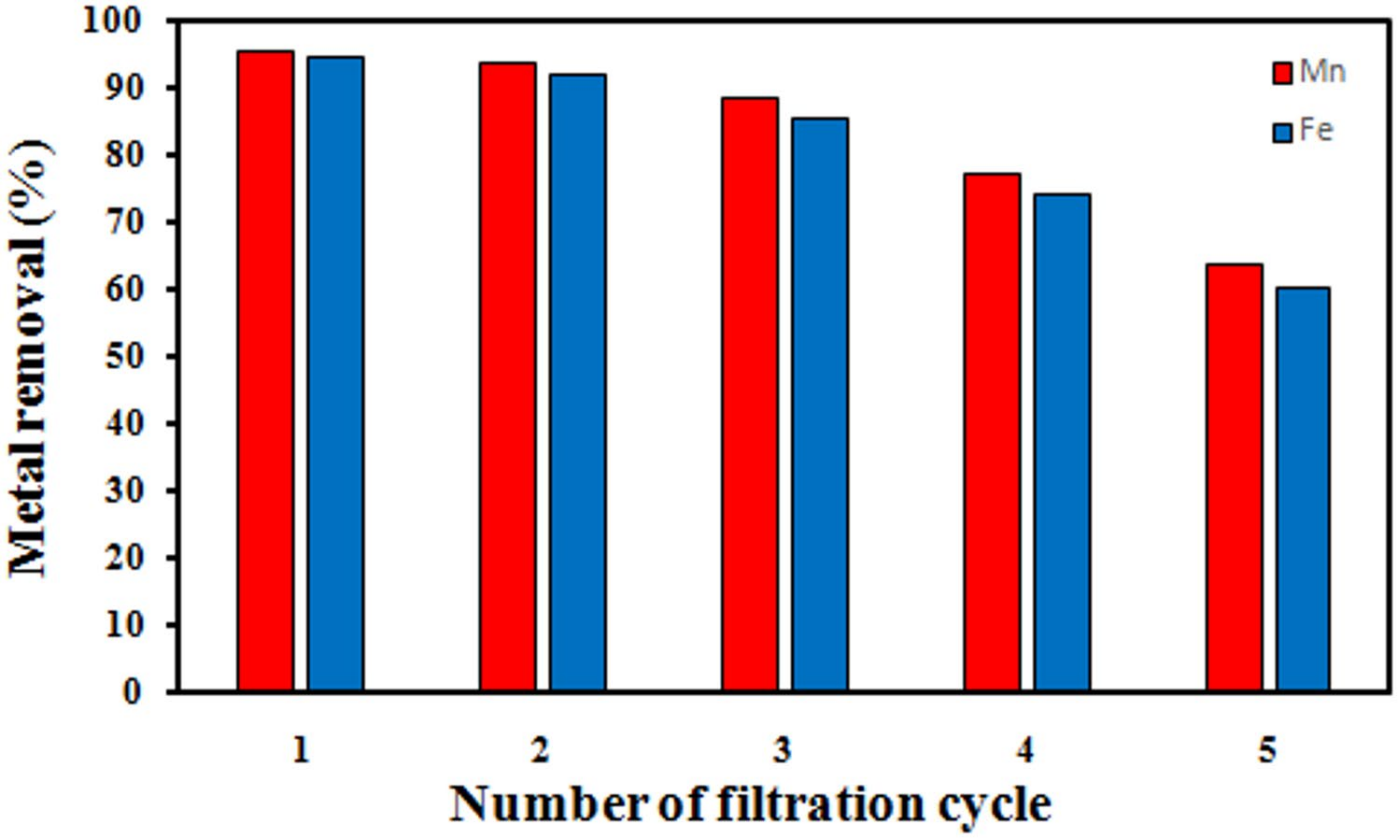
Percentage removal of metal ions at every filtration cycle.

During the initial filtration cycle, the developed membrane exhibited outstanding heavy metal ion removal efficiencies of 94.6% for Fe^2+^ and 95.5% for Mn^2+^. However, as the cycles progressed, the removal efficiency gradually declined, reaching approximately 50% after the fifth cycle, as presented in Fig. 23.

This observed reduction in performance can be attributed to the progressive saturation of active surface sites with metal ions and the partial blockage of the membrane pores, which limit the availability of active adsorption sites. Additionally, repeated washing and drying may cause partial detachment or structural rearrangement of the PVA/GONPs layer, further contributing to the decline in efficiency.

Despite this decrease, the membrane maintained reasonable removal efficiency even after multiple reuses, demonstrating its potential applicability for water purification. The results emphasize that periodic membrane regeneration or surface reactivation was essential to restore active sites and prolong long-term performance. Future work may therefore focus on developing effective regeneration techniques to mitigate the decline in removal efficiency, as well as testing the membrane against a broader spectrum of heavy metal ions to better assess its resilience in practical water treatment operations.

The effectiveness of composite membranes in removing various metal ions from water was contrasted in **Table (3).** It was evident that the combination of various materials in these composite membranes results in a range of metal ion removal efficiency. Previous studies that combined different polymer types with different composites have produced membrane removal capabilities of metal ions ranging from 60% to 86%^71–73^. However; the current study offers a novel approach that exhibits exceptional metal ion removal performance by applying PVA/GONPs to the PVDF membrane’s surface.

**Table 3:** The composite membranes’ relative effectiveness in removing metal ions.

No.	Membrane	Metals removed	Metal removal (%)	Ref.
1	Grapheneoxide–polyethersulphone (GO–PES)	Cu^2+^ Zn^2+^ Cd^2+^	About 65%–80% for all the metal ions	^74^
2	membranes of mesoporous silica nanocomposite functionalized with Polysulphone surfaces	Cd^2+^ Zn^2+^	91% 94%	^75^
3	Polyamide titanium dioxide (PA-TiO_2_)	Cu^2+^ Hg^2+^ Pb^2+^	86.89 ± 2% 77.01 ± 2% 83.42 ± 1.5%	^76^
4	Graphene oxide-manganese oxide (GO MnO_2_) nanohybrid sulphonated polyethersulphone (SPES) ultrafiltration (UF) membranes	Cu^2+^ Zn^2+^ Ni^2+^	81.1% 64.0% 67.4%	^77^
5	GO–PES (Grapheneoxide/polyethersulfone)	Mn^2+^ Fe^2+^	94% 93.6%	^78^
6	PVA/GO–PVDF	Mn^2+^ Fe^2+^	95.5% 94.6%	Present study

## Conclusions

In this study, GONPs was successfully synthesized using a modified Hummer’s process and applied to the surface of the PVDF membrane. Investigating GONPs- PVDF membranes for the elimination of HMIs, particularly iron (Fe) and manganese (Mn), from tainted water has yielded important new information and promising results. The successful synthesis of GONPs was confirmed by means of analysis methods such as scanning electron microscopy (SEM), FTIR spectroscopy, and XRD measurements. Membranes modified by the dip coating method were evaluated at different GONPs loadings using surface charge measurements, contact angle, and SEM. It was verified that the membrane’s surface contained GO nanoparticles. PVA helps to increase the effectiveness of GO nanoparticles on the PVDF membrane surface. Glutaraldehyde (GA) serves as a cross linker for PVA polymer chains, enhancing the thin film’s chemical and mechanical stability. Because of the oxygen-rich functional groups in GONPs, the membrane’s hydrophilic nature was verified by a reduced water contact angle. The modified membranes’ tensile strength rose from 3.58 to 6.15 MPa as the PVA/GO (0.1, 0.3, and 0.5 wt%) loading increased. The thin-film coating’s increasing GO nanoparticles content increased the heavy metal ion removal efficiency. As the thin-film GONPs loading rises, the nanoparticles begin to group together, decreasing the membrane’s porosity and permeate flow. During the initial filtration process, the GONPs-embedded PVDF membrane retained more than 95.5% of Mn^2+^ ions and 94.6% of Fe^2+^ ions. The nanoparticles remained stable over the course of five testing cycles, but their removal effectiveness declined. The filtering tests confirmed that GONPs-decorated PVDF membranes were more effective at removing Mn^2+^ and Fe^2+^ ions from contaminated water.

The effective production, incorporation, and use of GONPs-embedded PVDF membranes for the extraction of HMIs from water were shown in this work. This study advances water filtration technology, offers a potential method for removing heavy metal ions, and shows promise as an effective wastewater treatment tool.

## Data Availability

Data is provided within the manuscript files.
